# A novel pH-sensitive nanoparticles encapsulating anti-PD-1 antibody and MDK-siRNA overcome immune checkpoint blockade resistance in HCC via reshaping immunosuppressive TME

**DOI:** 10.1186/s13046-025-03396-6

**Published:** 2025-05-16

**Authors:** Hai Xu, Shuo Li, Yifan Liu, Yoon-Young Sung, Yong Zhou, Huikun Wu

**Affiliations:** 1 Department of Oncology, Huangjiahu Hospital of Hubei University of Chinese Medicine, Wuhan, China; 2 College of Traditional Chinese Medicine, Hubei University of Chinese Medicine, Wuhan, China; 3 Department of Clinical Laboratory, Institute of Translational Medicine, Renmin Hospital of Wuhan University, Wuhan, China; 4 Korea Institute of Oriental Medicine, Daejeon, Korea; 5 Hubei Key Laboratory of theory and application research of liver and kidney in traditional Chinese medicine, Affiliated Hospital of Hubei University of Chinese Medicine, Wuhan, China; 6 Hubei Shizhen Laboratory, Wuhan, China; 7 Hubei Institute of Traditional Chinese Medicine, Wuhan, China

**Keywords:** Hepatocellular carcinoma, Nanodrug, Midkine, Tumor-associated macrophages, Myeloid-derived suppressor cells, Immunotherapy

## Abstract

**Objective:**

Immunotherapy, notably the immune checkpoint blockade (ICB), has demonstrated significant promise in the management of diverse neoplasms. However, the PD-1 inhibitor has exhibited suboptimal objective response rates and did not achieve the primary endpoints in hepatocellular carcinoma (HCC) patients, primarily due to resistance to ICB fostered by the immunosuppressive tumor microenvironment (TME). To address ICI resistance and minimize adverse effects, we have engineered an innovative tumor-specific nanomedicine for the concurrent administration of aPD-1 and MDK-siRNA.

**Methods:**

Both in vitro and orthotopic HCC models were employed to investigate and establish the efficacy of the novel tumor-specific nanomedicine in overcoming the immunosuppressive TME. Specifically, the impact of the nanomedicine on the M2 polarization and polyamine metabolism within tumor-associated macrophages (TAMs) and myeloid-derived suppressor cells (MDSCs) was delineated. The immunomodulatory and antitumor effects, along with the side effects, of the nanomedicine integrating both aPD-1 and MDK-siRNA were assessed.

**Results:**

A dual pH-responsive nanomedicine was successfully fabricated to co-deliver MDK-siRNA and aPD-1. The nanomedicine achieved targeted drug delivery to tumors by engaging with circulating PD-1^+^ T cells and accompanying their migration into the tumor mass. Additionally, nanomedicine promoted efficient drug release within the acidic TME, deploying aPD-1 for ICI therapy and retaining MDK-siRNA-encapsulated nanomedicine to regulate TAMs and MDSCs synergistically. The synergistic application of MDK-siRNA and aPD-1, coupled with the efficient tumor-targeted drug delivery, potently suppressed M2 polarization and polyamine metabolism in TAMs and MDSCs, thereby overcoming the immunosuppressive TME and leading to significant therapeutic efficacy with minimal side effects in HCC.

**Conclusion:**

We have developed an innovative tumor-specific nanocarrier for the co-delivery of aPD-1 and MDK-siRNA. We validated that the synthesized nanomedicine (aPD-1-siRNA@NP) yielded highly effective treatment and minimal side effects in both in vitro and orthotopic HCC models. Our work presents a nanomedicine-based approach for targeted dual-drug delivery, achieving notable efficacy in the treatment of HCC.

**Supplementary Information:**

The online version contains supplementary material available at 10.1186/s13046-025-03396-6.

## Highlights

Aiming at reversing immunosuppressive TME and overcoming ICB resistance for HCC immunotherapy, a dual pH-sensitive nanodrug was successfully developed to facilitate T cell-mediated and tumor-targeted delivery of both aPD-1 and MDK-siRNA.

This novel nanodrug achieved remarkable ICB therapeutic efficacy with minimal side effects in HCC.

Our efforts offered a nanomedicine strategy for tumor-targeted dual-drug delivery to gain remarkable efficacy in HCC treatment.

**Supplementary Information:**

The online version contains supplementary material available at 10.1186/s13046-025-03396-6.

## Introduction

Hepatocellular carcinoma (HCC) constitutes one of the two predominant forms of primary liver malignancies, representing 70% of such cases, and in 2020, it was the sixth most commonly diagnosed cancer and the third highest cause of cancer-related fatalities on a global scale [1]. Internationally, liver cancer was a leading cause of cancer-related death, ranking within the top three in 46 nations and among the top five in 90 countries [2].

Immunotherapy, especially the targeting of the immune checkpoint blockade (ICB) against programmed cell death protein 1 (PD-1) or its ligand (PD-L1), has risen as a significant advancement in the treatment of HCC [3, 4]. Nevertheless, the objective response rate to anti-PD-1/PD-L1 immunotherapy in HCC is comparatively low, potentially due to resistance to ICB stemming from the immunosuppressive tumor microenvironment (TME) [5]. Consequently, there is an urgent requirement for innovative approaches that can concurrently tackle ICB resistance and mitigate adverse effects in HCC immunotherapy.

Myeloid cells associated with tumors, predominantly comprising tumor-associated macrophages (TAMs) and myeloid-derived suppressor cells (MDSCs), are instrumental in fostering an immunosuppressive TME, thereby facilitating tumor growth [6]. TAMs exhibit characteristics akin to M2-polarized macrophages under the influence of diverse signaling pathways and elements within the TME [7]. It is evident that M2 TAMs undermine T-cell cytotoxicity and enhance immunosuppression by secreting factors and chemokines that are immunosuppressive. MDSCs, which are pathologically activated myeloid cells originating from bone marrow myeloid progenitors, can not only generate a plethora of immunosuppressive factors that diminish T-cell cytotoxicity but also directly differentiate into M2 TAMs, sustaining a continuous supply of M2 TAMs [8]. Therefore, we hypothesize that the concurrent modulation of M2 TAMs and MDSCs to reverse the immunosuppressive TME could present a promising strategy to combat ICB resistance.

Midkine (MDK), a heparin-binding growth factor, is linked to anti-apoptotic, mitogenic, angiogenic, and chemoresistant properties [9]. Comprising two domains, each with three antiparallel β-strands and multiple heparin-binding consensus sequences, MDK interacts with heparan sulfate and chondroitin sulfate, facilitating the formation of molecular complexes with proteoglycans [10]. Suggested as a candidate biomarker for HCC, MDK expression is reportedly elevated in HCC patients versus healthy controls [11]. Research has indicated that small interfering RNA targeting the MK gene (MDK-siRNA) enhances HCC cell sensitivity to sorafenib [12, 13].

During the past several decades, nanocarrier-mediated drug delivery has shown promise in enhancing therapeutic efficacy and minimizing adverse effects [14, 15, 16]. In this study, leveraging the ability of circulating PD-1 + T cells to bind to the aPD-1 and migrate chemotactically toward tumors, we engineered a polymeric nanomicelle encapsulating MDK-siRNA, decorated with aPD-1 on its surface to engage circulating PD-1 + T cells for targeted drug delivery to tumors. An acid-labile linkage sensitive to TME was incorporated between aPD-1 and the nanomicelle, enabling swift antibody shedding in the TME. This releases aPD-1 for ICB and leaves behind the MDK-siRNA-encapsulated nanomicelle, which is readily internalized by M2 TAMs and MDSCs. Following lysosomal drug release, MDK-siRNA is anticipated to neutralize the immunosuppressive TME by modulating both M2 TAMs and MDSCs, thereby augmenting ICB efficacy in HCC immunotherapy in conjunction with aPD-1.

## Methods

### Preparation of nanodrugs

The polymer was crafted through a multi-step process, and the successful fabrication was confirmed by proton nuclear magnetic resonance (1 H NMR) and Fourier transform infrared (FTIR) spectroscopy (Supplementary Materials). The pH-responsive copolymer self-assembled into a nanomicelle, with MDK-siRNA encapsulated within the hydrophobic core. Subsequently, aPD-1 was affixed to the surface of the prepared nanomicelle to yield the final nanodrug (aPD-1-siRNA@NP). The encapsulation efficiency of siRNA was assessed using a Ribogreen fluorometric assay. The nanodrug’s morphology was scrutinized using transmission electron microscopy (TEM).

### Antibody labeling and release

aPD-1-decorated nanodrug was incubated with Alexa Fluor^®^ 488 IgG (goat anti-rat aPD-1) at pH 7.4 for 6 h and followed by an ultrafiltration to remove the free secondary antibodies. Then the labeled nanodrug was incubated at various pH values (pH 7.4 and 6.5) for different times (0.5, 1, 2, 3, 4, 6, 8, 12 and 24 h). The free labeled antibodies were removed by ultrafiltration. The fluorescence intensity of Alexa Fluor^®^ 488 was determined at the emission wavelength of 525 nm.

In vitro aPD-1 release assay found that nearly 60% aPD-1 was released from nanodrug at pH 6.5 within 4 h. The results demonstrated that the antibody could be efficiently released responded to TME acidity, which was essential for aPD-1 to activate the cytotoxic T cells. Moreover, the aPD-1 release induced a potential reversal of nanodrug, which might facilitate the endocytosis of nanodrug by TAMs and MDSCs.

### T-cell binding in vitro

Lymphocytes were extracted from the spleens of C57BL/6 mice harboring tumors using a commercial separation kit (Miltenyi Biotec, Germany). The lymphocytes were cultivated in RPMI1640 medium (Gibco, USA) enriched with 10% Fetal bovine serum (FBS) at a concentration of 1 × 10^7^ cells/mL. The lymphocytes were exposed to aPD-1-adorned nanodrug at distinct time intervals (12 and 24 h) at a pH of 7.4 to facilitate binding. To further assess the disengagement of nanodrug from lymphocytes, the medium’s pH was altered to 6.5, and the lymphocytes were incubated for various durations (12 and 24 h) to enable detachment. The lymphocytes were labeled with Alexa Fluor 647 anti-mouse CD8 antibody and a secondary antibody Alexa Fluor 488 targeting aPD-1 for 1 h, while the nuclei were counterstained with Hoechst for 15 min. Ultimately, cellular imagery was obtained through Confocal Laser Scanning Microscopy (CLSM) (Zeiss LSM880, Germany).

### Cellular uptake of the nanodrug

Primary macrophages were derived from the femurs of C57BL/6 mice with tumors and activated by 20 nM macrophage colony-stimulating factors (M-CSF). These macrophages were subsequently stimulated by 25 nM IL-4 to induce M2 polarization. MDSCs were harvested from the spleens of C57BL/6 mice with tumors utilizing a commercial separation kit (Miltenyi Biotec). M2 macrophages or MDSCs were plated in 35 mm confocal dishes at a concentration of 1 × 10^4^ cells per well and incubated overnight, followed by treatment with aPD-1-adorned nanodrug at pH levels of 7.4 and 6.5 for 4 hours. For CLSM observation, cells were fixed with 4% paraformaldehyde and stained with 4’,6-diamidino-2-phenylindole (DAPI) for nuclear identification. For flow cytometry analysis, M2 macrophages or MDSCs were plated in 12-well plates at a concentration of 1 × 10^6^ cells per well and incubated overnight, then treated with aPD-1-adorned nanodrug at pH levels of 7.4 and 6.5 for 1, 2, and 4 hours. Subsequently, cells were harvested, resuspended in PBS, and analyzed using a flow cytometer.

### Immunofluorescence staining in vitro

M2 macrophages were seeded on circular glass slides on a 24-well plate and cultured with or without MDK-siRNA for 24 h. The cells were then fixed with 4% paraformaldehyde, permeabilized with 0.3% Triton X-100, and incubated with 5% bovine serum albumin (BSA) for 30 min at 37 °C. After overnight incubation with the primary antibodies at 4 °C, the cells were further incubated with the corresponding secondary antibody IgG Alexa Fluor 488 or IgG Alexa Fluor 594 for 1 h at 37 °C. The cells were stained with DAPI for nuclear localization. Finally, the glass slides with macrophages were collected and imaged using CLSM.

### HCC animal model and treatment

The orthotopic HCC model was established in male C57BL/6 mice (6 weeks old). Briefly, after a midline incision in the mouse abdomen, 1 × 10^6^ Matrigel-mixed Hepa1-6 cells (authenticated cell line) were injected into the liver. Subsequently, HCC mice were divided into various groups based on the principle of randomization.

### Biodistribution of nanodrugs

The nanodrug was tagged with DiD (0.1 mol%) and administered intravenously to HCC-bearing mice via the tail vein at an siRNA dosage of 0.5 mg/kg. At 4–24 h post-administration, mice were euthanized, and tumors as well as major organs were harvested. Biodistribution was visualized using a FluorVivo™ IVIS in vivo imaging system (INDEC Biosystems). To trace the siRNA cargo, Alexa Fluor 488-labeled siRNA was encapsulated into the nanomicelle. Fluorescence intensity in various organs was quantified using Image J software (NIH, USA).

### Immunofluorescence staining for tumor sections

Tumor sections were incubated with a CD8 antibody overnight at 4 °C. After triple washing with PBS, the sections were incubated with the corresponding secondary antibodies of IgG Alexa Fluor 594 at room temperature for 60 min. Finally, the sections were counterstained with DAPI and examined under CLSM. The tumor sections were stained with DAPI for nuclear localization and observed by CLSM.

### Histological and immunohistochemical analyses

Hematoxylin-eosin (H&E) staining was performed to assess the morphology of tumor tissues and major organs of HCC mice. Tissues were fixed in 4% paraformaldehyde, embedded in paraffin, and sectioned. The tissue sections were deparaffinized, hydrated, and stained with hematoxylin and eosin following standard protocols. For immunohistochemical staining, tissue sections were deparaffinized, rehydrated, and incubated with 3% hydrogen peroxide to quench endogenous peroxidase activity. After antigen retrieval in 10 mM sodium citrate buffer and blocking with 5% BSA solution, the tissue samples were incubated with primary antibodies overnight at 4 °C and corresponding HRP-conjugated secondary antibodies for 1 h at 37 °C. The tissue sections were then stained with a diaminobenzidine (DAB) kit. After counterstaining with hematoxylin, the tissue sections were mounted with neutral balsam medium.

### T cell-binding in vivo

HCC mice were injected via the tail vein with a PD-1-adorned nanodrug. Mice blood was collected at various time points (0, 12, and 24 h), and blood T cells were isolated and purified. The T cells were incubated with a CD8 antibody and stained with Hoechst 33,342, respectively. The T cells were then observed under CLSM. To evaluate the aPD-1-blocked CD8 + T cells at the tumor site, tumor-infiltrating T cells were isolated using a commercial T-cell isolation kit. These cells were stained with an anti-CD8 antibody and a secondary antibody IgG-APC-Cy7 (aPD-1) for 1 h. Afterward, the cells were washed and analyzed using a Flow Cytometer.

### MRI scan in vivo

Prior to treatment initiation and on days 7 and 14 post-first treatment, MRI scans were conducted to assess tumor growth in orthotopic HCC mice. Orthotopic tumors were detected using a clinical 3.0 T MRI system. Tumor volume was calculated using the formula tumor volume = 0.5 × length × width².

### Biosafety of nanodrug

Acute and chronic toxicity studies were performed to ascertain the biosafety of the nanodrug. In the acute toxicity study, HCC-bearing mice were administered the nanodrug intravenously at an siRNA dose of 3 mg/kg. At 24 h post-administration, serum samples were collected, centrifuged for 30 min (1200 g, 4 °C), and plasma was separated. Hepato-renal function markers were measured, including alanine aminotransferase (ALT), aspartate aminotransferase (AST), albumin, and total bilirubin (TBIL). Gene expression of pro-inflammatory cytokines IL-6, TNF-α, and IFN-γ was evaluated in tumor tissues 24 h post-administration.

In the chronic toxicity study, serum was collected from mice at the end of the repeated treatment with the nanodrug following the same schedule. Hepato-renal function markers were measured in plasma and normalized to HEPES buffer-treated mice. A histopathological examination of the liver was also conducted after chronic treatment to detect any potential injury.

### Statistical analyses

All processing and analysis procedures were executed in R software version 4.1.3.

## Results

### MDK-siRNA suppressed M2 macrophage polarization

**Fig. 1:**
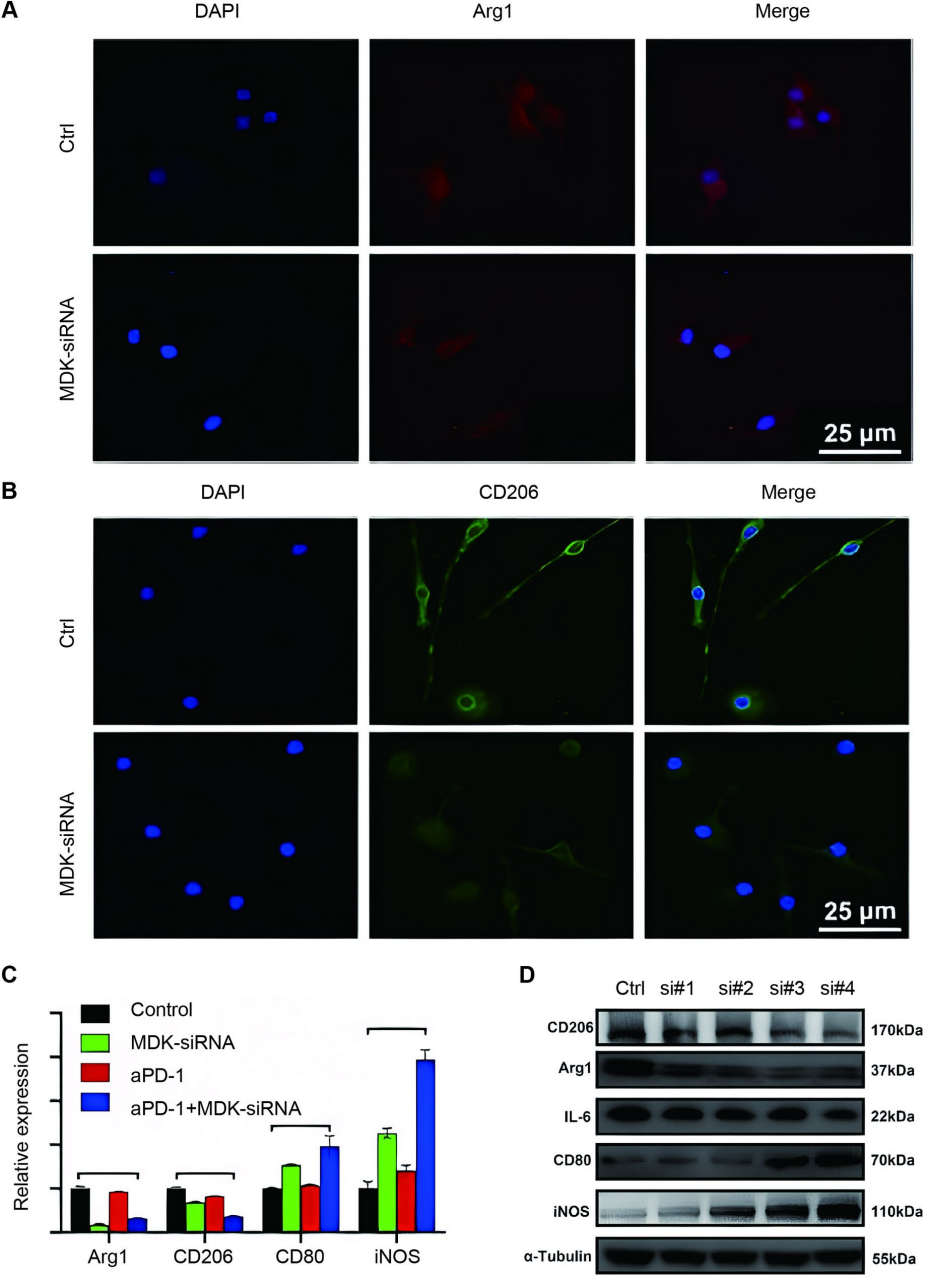
Inhibition of M2 Macrophage Polarization by MDK-siRNA. (**A-B**) MDK-siRNA suppresses M2 polarization markers (Arg1 and CD206) in macrophages. (**C**) Quantitative RT-PCR analysis of Arg1, CD206, CD80, and iNOS mRNA levels in macrophages. (**D**) Western blot analysis of Arg1, CD206, CD80, iNOS, and IL-6 protein expression in macrophages treated with various MDK-siRNA constructs

In this study, macrophages activated by IL-4 served as a canonical model for M2-TAMs [17]. We investigated the potential of MDK-siRNA to regulate the M2 phenotype in the IL-4-activated Raw264.7 macrophage cell line and primary macrophages derived from the femurs of mice. MDK-siRNA substantially inhibited M2 polarization (with Arg1, CD206, and IL-6 serving as indicative markers) and enhanced M1 polarization (marked by CD80, iNOS, and TNF-α) in these macrophages. (Fig. 1 and Fig. S21-S22).

### MDK-siRNA reactivated CD8^+^ T cells via regulating TAMs and MDSCs

**Fig. 2:**
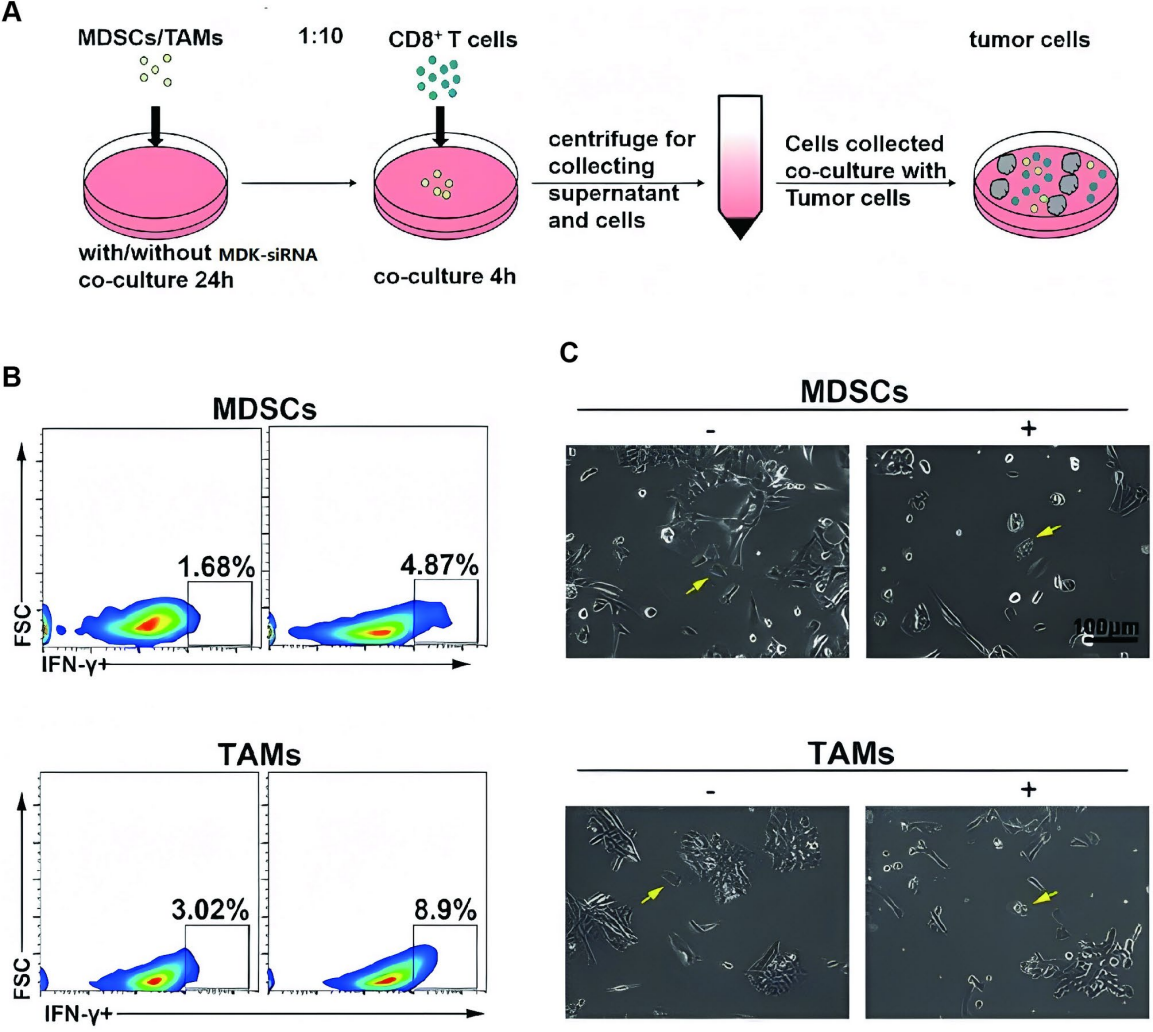
MDK-siRNA-Mediated Reactivation of CD8 + T Cells through Regulation of TAMs and MDSCs. (**A**) Schematic representation of the coculture system. (**B**) Flow cytometry quantification of CD8 + T cells within the coculture system. (**C**) Morphological changes and viability assessment of Hep1-6 HCC cells in the coculture system with or without MDK-siRNA treatment, as evaluated by CCK-8 assays

Subsequently, we assessed the effects of MDK-siRNA-treated M2-TAMs/MDSCs on T-cell stimulation and tumorigenesis. A co-culture model was developed, comprising splenic CD8 + T cells from immunocompetent rodents, MDSCs/M2 macrophages, and Hepa1-6 HCC cells [18]. Flow cytometric analysis indicated that pre-treatment of M2-TAMs/MDSCs with MDK-siRNA markedly enhanced the proportion of IFN-γ + cells and IFN-γ output in the co-culture environment (Fig. 2 and Fig. S23-S24). Afterward, the cytotoxic impact of CD8^+^ T cells on tumors was appraised using the CCK-8 assay (Fig. 2C). The suppressive effect of MDSCs or M2-TAMs on the cytotoxicity of CD8^+^ T cells was significantly mitigated by MDK-siRNA therapy (Fig. S25). Hence, we established that MDK-siRNA could counteract the suppressive effects of M2 TAMs and MDSCs on CD8 + T cells.

### In vitro T-Cell engagement and cellular internalization of nanomedicine

**Fig. 3:**
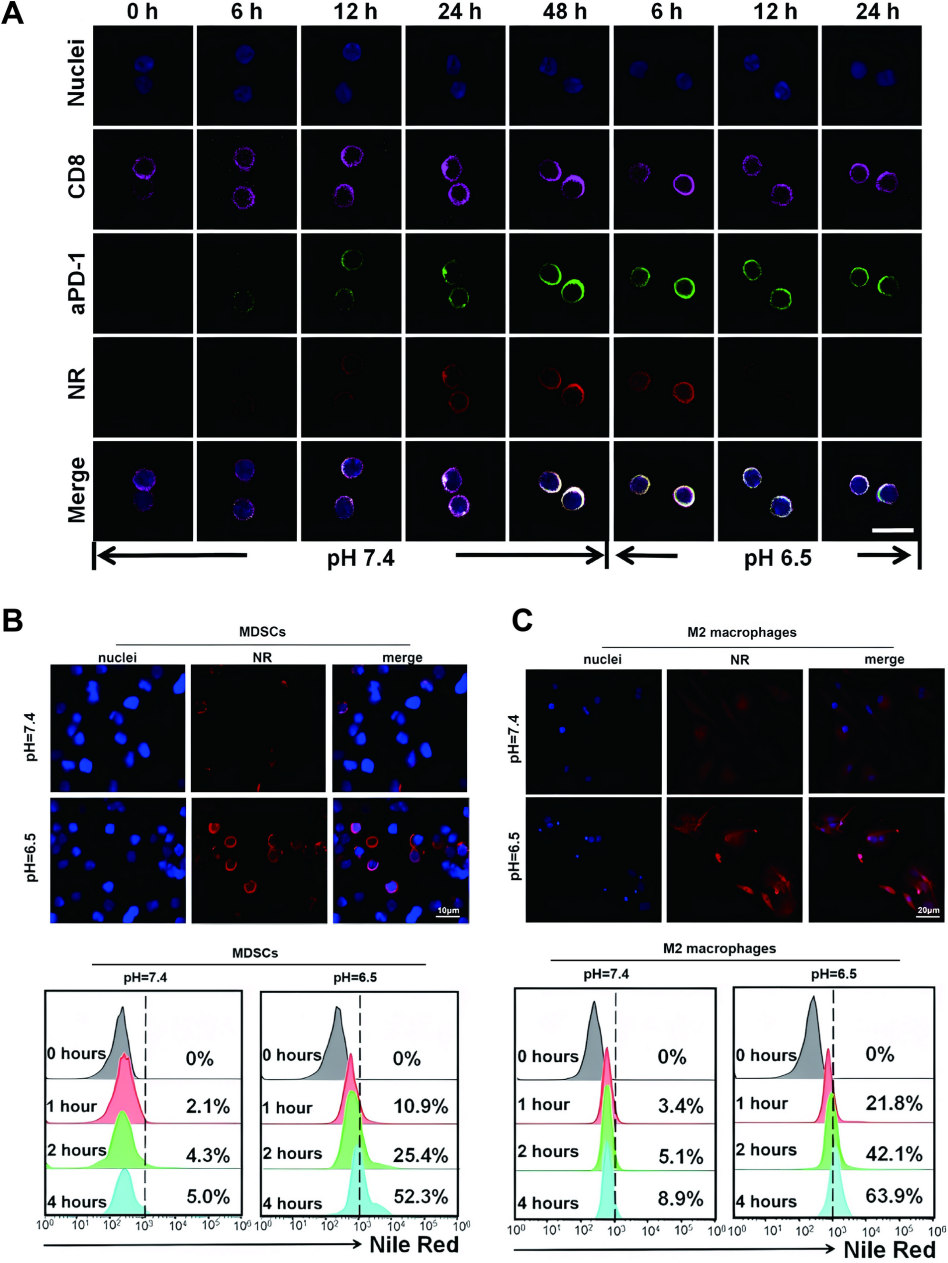
In Vitro T-Cell Binding and Cellular Uptake of Nanodrug. (**A**) CD8 + T cells (purple Alexa Fluor 647) targeted in in vitro binding studies; aPD-1 labeled with green Alexa Fluor 488, and MDK-siRNA represented by hydrophobic red fluorochrome NR for fluorescence visualization. CLSM imaging reveals nanodrug binding to PD-1 + T cells at pH 7.4 and 6.5. (**B-C**) CLSM imaging and flow cytometry assessment of nanodrug cellular uptake in M2 macrophages and MDSCs at pH 7.4 and 6.5

CD8 + T cells (purple Alexa Fluor 647) were targeted in the in vitro binding study to demonstrate the activation of nanomedicine on antitumor immunity through effective engagement with CD8 + T cells [19]. At pH 7.4, simulating the physiological bloodstream environment, the fluorescence intensity of both aPD-1 (green Alexa Fluor 488) and MDK-siRNA (hydrophobic red fluorescence of NR) escalated over incubation time and was predominantly localized on the surface of CD8 + T cells (Fig. 3A), signifying successful nanomedicine binding to CD8 + T cells via PD-1-aPD-1 interaction rather than internalization. At pH 6.5, reflecting TME acidity, the red fluorescence signals on the cell surface diminished rapidly with incubation time, indicating the detachment of NR-tagged nanomicelles from CD8 + T cells (Fig. S26).

Thereafter, MDSCs and M2 macrophages were designated as target cells for the in vitro drug uptake study [20]. CLSM observations revealed that both MDSCs and M2 macrophages exhibited distinct NR red fluorescence in the cytoplasm following a 4-hour incubation at pH 6.5, indicative of efficient internalization of nanomedicine (Fig. S27). In contrast, cells incubated at pH 7.4 exhibited minimal drug uptake (Fig. 3B&C). These findings were quantitatively validated by flow cytometry analysis. The results suggest that nanomedicine is readily internalized by MDSCs and M2 macrophages, potentially due to the surface charge transition from negative to positive post initial aPD-1 release and the elevated endocytic activity of MDSCs and macrophages.

The NF-κB signaling pathway is pivotal in modulating tumor immune responses [21]. Consequently, MDK-siRNA delivered via pH-sensitive nanomedicine is anticipated to impede the NF-κB signaling pathway by preventing p65 translocation to the nucleus. Immunofluorescence staining for pho-p65 revealed that aPD-1-siRNA@NP treatment elicited the faintest green fluorescence in the nucleus of M2 TAMs and the faintest purple fluorescence in the nucleus of MDSCs (Fig. S28), suggesting that nanomedicine potently inhibits NF-κB phosphorylation. Western blot assays confirmed that aPD-1-siRNA@NP treatment significantly downregulated pho-p65 protein expression in both M2 TAMs and MDSCs, irrespective of LPS pre-stimulation (Fig. S29). Similarly, PD-L1 protein expression in M2 TAMs and MDSCs treated with aPD-1-siRNA@NP was also effectively curtailed. These findings imply that nanomedicine (aPD-1-siRNA@NP) not only obstructs the PD-1 immune checkpoint but also significantly suppresses the NF-κB pathway in both M2 TAMs and MDSCs.

### In vivo binding of the nanodrug to CD8^+^ PD-1^+^ T cells

**Fig. 4:**
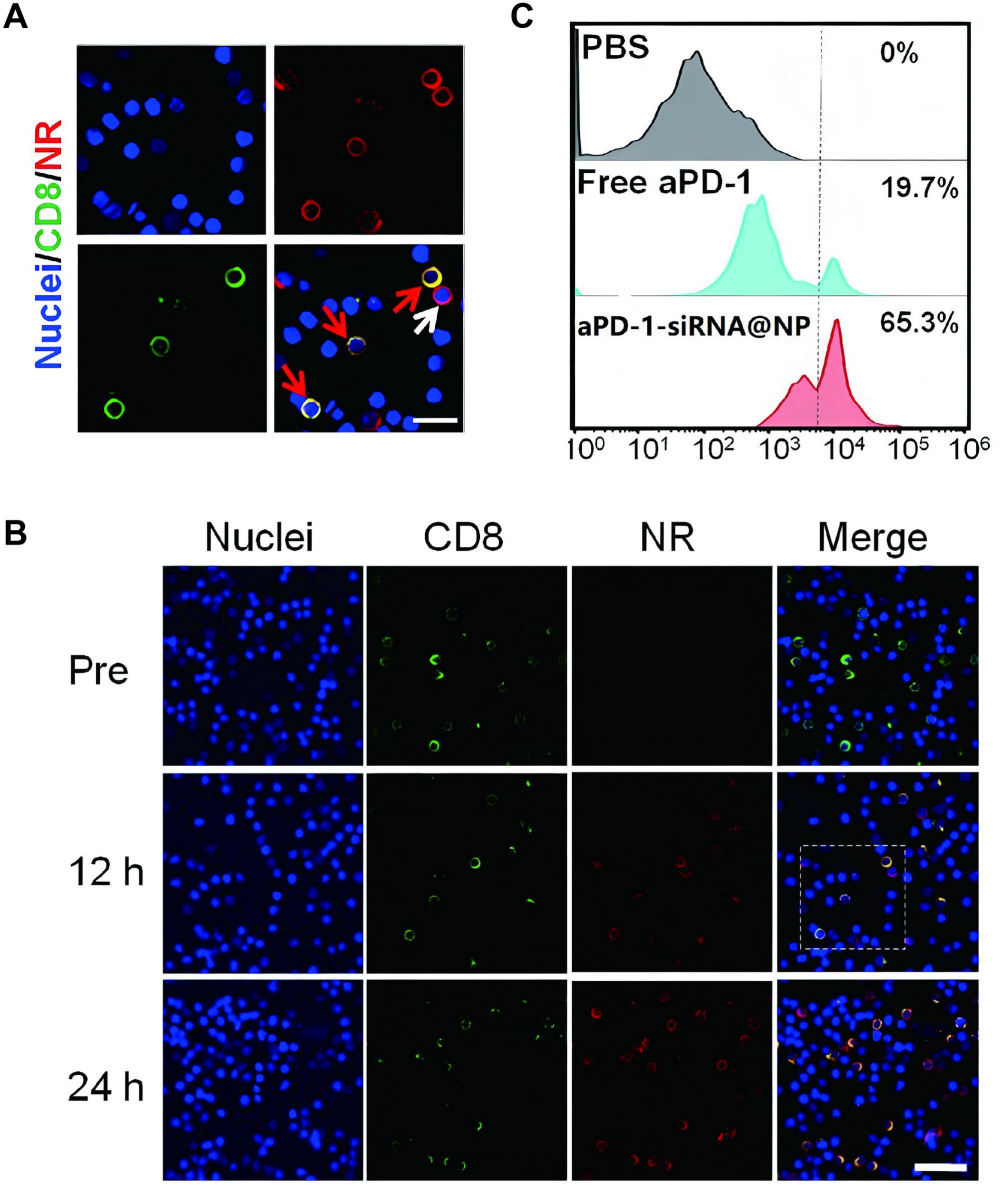
In Vivo Binding of Nanodrug to CD8 + PD-1 + T Cells. (**A-B**) CLSM images illustrate the binding of nanodrug to PD-1^+^ T cells at 12 h after tail vein injection. Red arrows indicate nanodrug-bound PD-1^+^CD8^+^ T cells, and white arrow illustrates a PD-1^+^CD8^−^ T cell attached with nanodrug. (**C**) Flow cytometry analysis of CD8 + T cells (defined as CD3 + CD4 − CD8 + T cells) blocked by Alexa Fluor 555-labeled aPD-1 in tumors at 3 days following tail vein injection of PBS, free PD-1, and nanodrug

To assess the in vivo binding affinity of the nanodrug to CD8 + PD-1 + T lymphocytes, these cells were extracted from the blood and examined using Confocal Laser Scanning Microscopy (CLSM) 24 h post-injection of the nanodrug into rodents via the tail vein [22]. As depicted in Fig. 4, the majority of CD8 + T cells (visualized with green Alexa Fluor 488) were adorned with red fluorescent NR (indicated by red arrows), signifying efficient nanodrug binding to CD8 + T cells. Furthermore, 24 h post-injection, tumor-infiltrating T lymphocytes were harvested for quantitative flow cytometry assessment. Within the mice administered free aPD-1, only 17.1% of the tumor-infiltrating CD8 + T cells were obstructed by aPD-1 (identified as APC-Cy7 positive), whereas the APC-Cy7-positive cell population escalated to 54.3% in mice subjected to nanodrug treatment. These findings suggest that the nanodrug potently inhibits the PD-1-PD-L1 interaction.

To substantiate that the aPD-1-adorned nanodrug, sensitive to TME acidity (pH 6.5), could be effectively transported to the tumor by PD-1 + T cells, we ascertained the colocalization of PD-1 with aPD-1 through CLSM analysis. As illustrated in Fig. S30, the red fluorescence of PD-1 on the T cell membrane coincided with the green fluorescence of aPD-1. To probe the tumor deposition of nanomicelles, the nanodrug was injected into mice harboring primary HCC via the tail vein, and the animals were sacrificed 24 h post-injection to procure tumor sections. CD8 + T cells were labeled with an Alexa Fluor 647-conjugated anti-CD8 antibody (displaying purple fluorescence), and aPD-1 was marked with a secondary antibody IgG Alexa Fluor 488 (exhibiting green fluorescence) for CLSM visualization. The purple T cell membrane fluorescence coincided with the green aPD-1 fluorescence, while the surrounding red fluorescence was distinct from aPD-1 and the CD8 + T cells. These outcomes furnished compelling evidence that the aPD-1-adorned nanodrug could be conveyed to the tumor and subsequently liberated by the tumor-infiltrating CD8 + PD-1 + T cells.

### Nanodrug reversed the immunosuppressive TME by regulating M2 TAMs and MDSCs

**Fig. 5:**
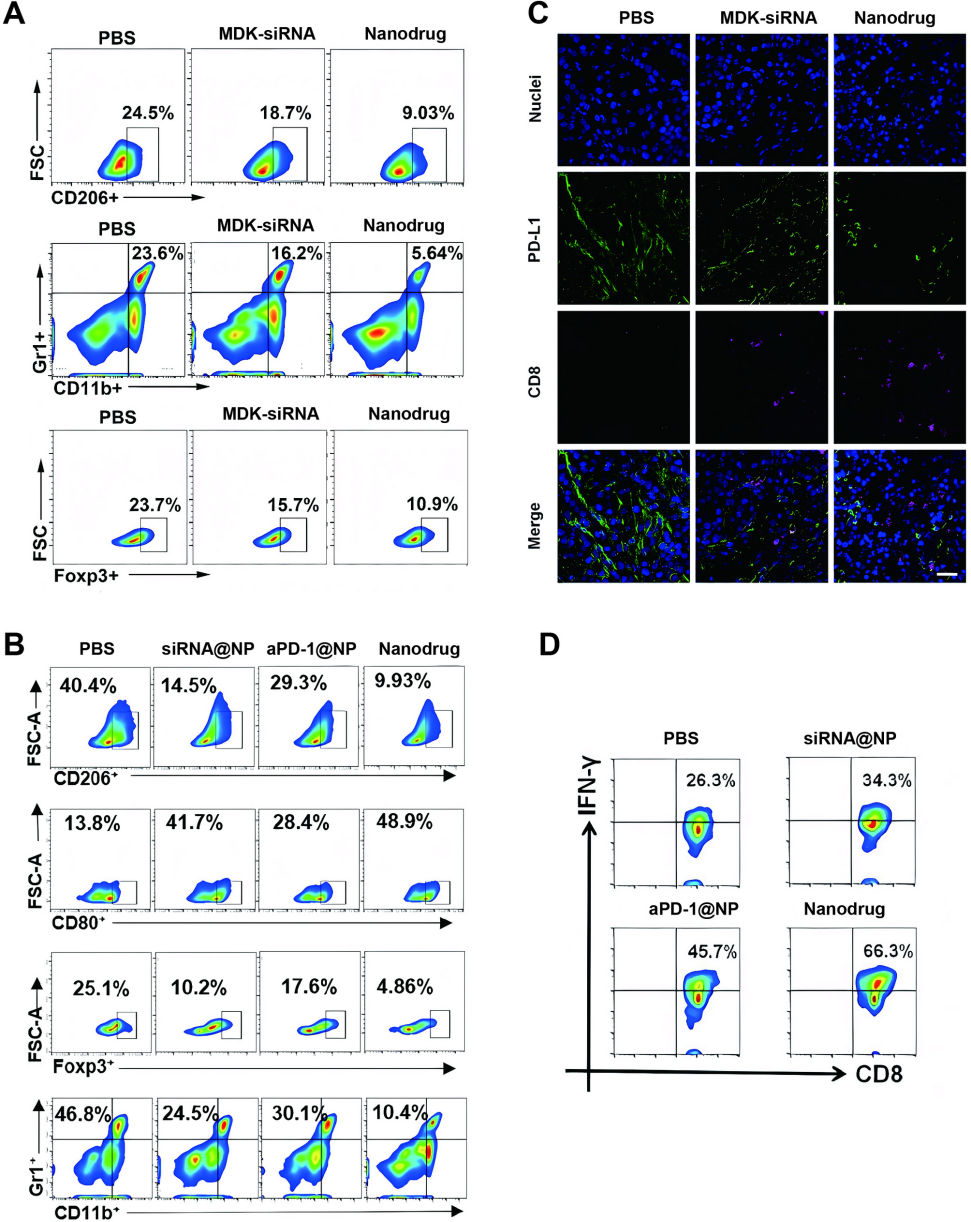
Reversal of Immunosuppressive TME by Nanodrug via Regulation of M2 TAMs and MDSCs. (**A-B**) Flow cytometry quantification of M2 macrophages (CD206+, within CD11b + F4/80 + cells), MDSCs (Gr1+, within CD11b + cells), and Treg cells (Foxp3+, within CD3 + CD4 + cells) in Hep1-6 tumor tissues. (**C**) Immunofluorescence analysis of Hep1-6 tumor sections for CD8 + T cell infiltration and PD-L1 expression at 15 days of in vivo study. CD8 + T cells are labeled with Alexa Fluor 647 (purple fluorescence), PD-L1 with Alexa Fluor 488 (green fluorescence), and cell nuclei with DAPI (blue fluorescence). (**D**) Flow cytometry analysis of CD8 + IFN-γ + T cells in Hep1-6 tumors at 15 days of in vivo study (defined as CD3 + CD4 − CD8 + T cells)

M2 TAMs, MDSCs and regulatory T cells (Tregs) behave as main immunosuppressive cells which cause tumor immune escape [23]. In vivo flow cytometry determined that MDK-siRNA was capable of suppressing M2 polarization of TAMs and reducing the proportion of MDSCs (CD11b + Gr+) in mice bearing orthotopic HCC (Fig. 5A). In addition, we further found that MDK-siRNA significantly decreased the proportion of Treg cells (CD4^+^Foxp3^+^). Moreover, the counteraction of MDK-siRNA against the immunosuppressive TME was significantly strengthened in the group receiving nanodrug, which could be attributed to the enhanced cell uptake of nanodrug facilitated by the TME acidity. Taken together, nanodrug delivered by nanocarrier displayed a great in vivo performance to counteract the immunosuppressive TME of HCC by simultaneously regulating M2 TAMs and MDSC.

The CLSM images of immunofluorescence staining in tumor center revealed consistent results (Fig. 5B). The animals treated with PBS showed hardly detectable tumor infiltration of CD8^+^ T cells, and the MDK-siRNA treatment only slightly increased the tumor infiltration of CD8^+^ T cells. In contrast, the nanodrug treatment much more effectively increased the tumor infiltration of CD8^+^ T cells. Meanwhile, the nanodrug treatment resulted in the lowest expression level of PD-L1 in the tumor tissue, implying a greatly impaired immune checkpoint PD-1/PD-L1 axis. The increased CD8^+^ T cells in the tumor tissue via nanodrug treatment may partially be due to a reactivation upon the PD-L1 suppression. These results demonstrated that the NF-κB pathway inhibition by nanodrug in vivo lowered the tumor infiltration of M2 TAMs and MDS, which in turn promoted the recruitment of CD8^+^ T cells to the tumor.

To verify the role of nanodrugs in conquering ICB resistance for HCC treatment, mice bearing orthotopic HCC were administered different therapies (siRNA@NP, aPD-1@NP, or the combination of MDK-siRNA and aPD-1, i.e., aPD-1-siRNA@NP) via tail vein injection [24]. As expected, the group receiving aPD-1@NP displayed a slightly increased proportion of M1 TAMs (CD80 as marker) and CD8^+^ T cells and a mildly reduced proportion of M2 TAMs (CD206 as marker), Tregs and MDSCs in the tumor tissues (Fig. 5C). Inspiringly, aPD-1-siRNA@NP showed the most effective modulation on the HCC immune microenvironment, as evidenced by the highest proportion of M1 TAMs and CD8^+^ T cells and the lowest proportion of M2 TAMs, Tregs and MDSCs in the tumor tissues. These in vivo results verified that codelivery of MDK-siRNA and aPD-1 jointly fostered an antitumor immune microenvironment.

It is known that CD8^+^ T cells secrete cytokines interferon-γ (IFN-γ) and tumor necrosis factor-α (TNF-α) to kill tumor cells. However, this function was often impaired by the immune checkpoint PD-1/PD-L1 axis in the TME, which makes the blockage of PD-1/PD-L1 axis with aPD-1 a promising strategy in tumor immunotherapy [25]. Thus, flow cytometry assay was performed to evaluate the activation of CD8 + T cells via quantifying the CD8 + T cells expressing IFN-γ (Figs. 5D and 31). The ratio of CD8^+^ T cells with intracellular IFN-γ expression increased in the siRNA@NP and aPD-1@NP treatments as compared with the PBS treatment. More importantly, the aPD-1-siRNA@NP treatment resulted in the highest ratio of IFN-γ-expressing cells, which indicated a combination effect of MDK-siRNA and aPD-1 on the activation of CD8 + T cells.

### Nanodrug regulated TME and achieved enhanced therapeutic outcome in HCC

**Fig. 6:**
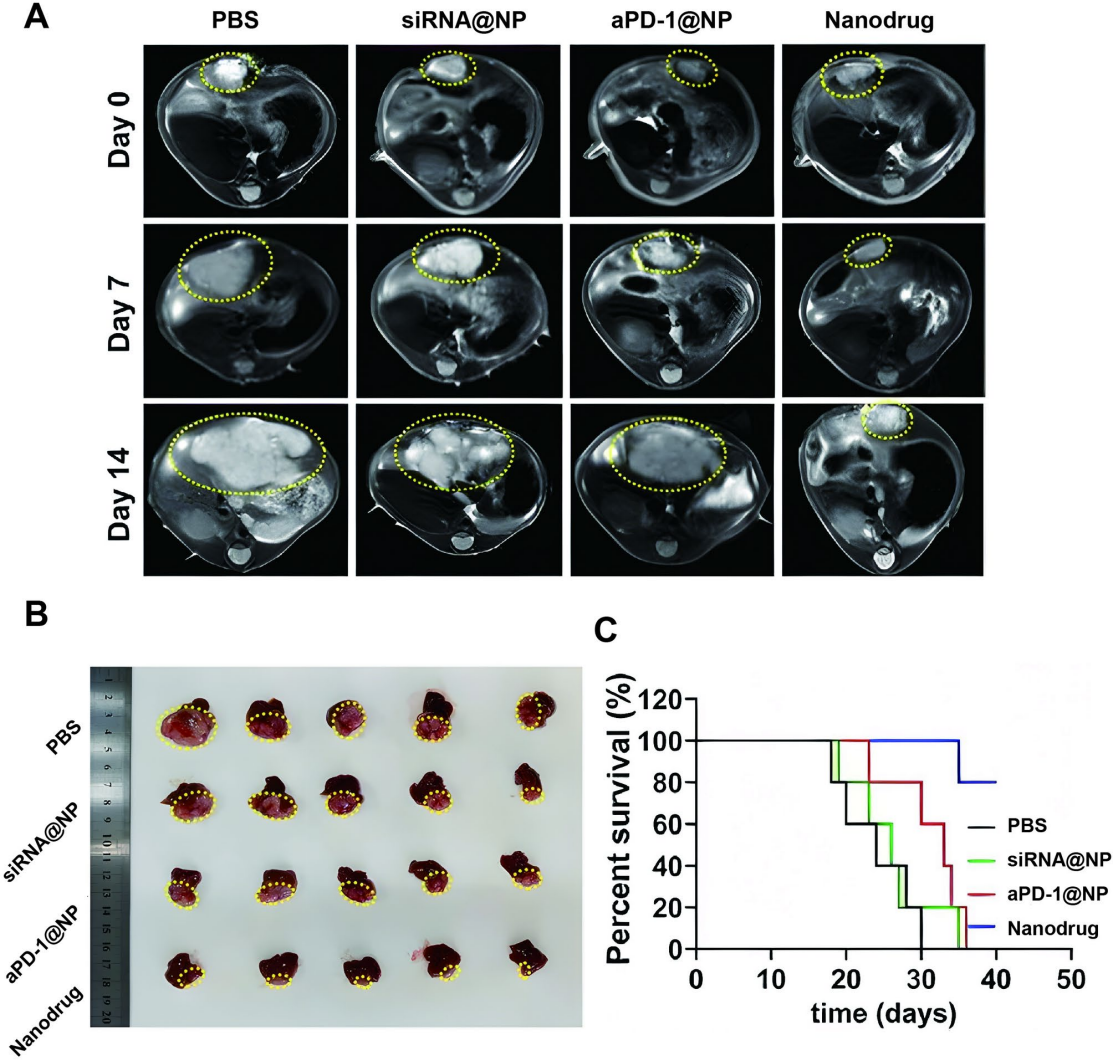
Modulation of TME and Enhanced Therapeutic Efficacy of Nanodrug in HCC. (**A**) Tumor growth monitoring via T2WI MRI at various time points. (**B**) Overview of orthotopic HCC across different treatment groups. (**C**) Survival analysis of orthotopic HCC mice

Next, the antitumor effect of nanodrug was further evaluated. On one hand, compared with the monotherapy of siRNA@NP or aPD-1@NP, the combination therapy of aPD-1-siRNA@NP exhibited the most potent suppression on tumor growth as determined by the MRI scan and anatomical observation, and brought the longest survival for the animals until the end point of observation (Fig. 6). Pharmacokinetic parameters of the nanodrug following intravenous.

administration to mice was shown in Table S1. Furthermore, histo-pathological examinations of the major organs following nanodrug treatment did not reveal any signs of injury or damage (Fig. S32).

## Discussion

Immunotherapy, particularly the ICB (e.g., PD-1 inhibitor), has shown great potential for treating various malignant tumors [26]. Nevertheless, PD-1 inhibitors obtained poor objective response rates (15-20%) in phase I/II trials and failed to meet the primary end points in phase III trials for patients with HCC, which was primarily attributable to ICB resistance induced by the immunosuppressive TME, as well as to drug discontinuation caused by serious immune-related adverse events. Thus, novel anti-HCC strategies that can simultaneously overcome ICB resistance and reduce side effects are urgently needed. Aiming at overcoming the aforementioned dilemma, we develop a novel tumor-targeted nanocarrier for the combined delivery of aPD-1 and MDK-siRNA. We confirmed the formed nanodrug (aPD-1-siRNA@NP) achieved a highly effective therapy and minimal side effects in vitro and in an orthotopic HCC model.

In this study, we introduced a tailor-made block copolymer to endow aPD-1-siRNA@NP with the property of dual pH-sensitive drug release. In response to the TME’s weak acidity (pH 6.5), aPD-1-siRNA@NP delivered to the tumor tissue by either recruiting nanodrug-bound PD-1 + T cells triggers the first-stage drug release in TME to provide aPD-1 for blocking the PD-1-PD-L1 axis to activate the tumor-killing cytotoxic T cells [27]. Meanwhile, the first-stage drug release also leads to a surface charge reversal from negative to positive, leaving a positively charged MDK-siRNA-encapsulated nanomicelle, which allows an easier drug internalization into M2 macrophages and MDSCs. Next, after entering the cytoplasm, the nanodrug triggers the rapid second-stage drug release to provide MDK-siRNA for dually inhibiting M2 macrophages and MDSCs to reverse immunosuppressive TME [28]. Taken together, we offered a nanomedicine strategy for tumor-targeted dual-drug delivery to gain remarkable efficacy in HCC treatment.

We were challenged by multiple obstacles facing the in vivo delivery of gene therapies to HCC [26]. First, the delivery vehicle should exhibit good pharmacokinetics and have sufficient circulation time following systemic administration to avoid rapid clearance by the first-pass organs, such as liver and spleen or the reticuloendothelial system [29]. Second, the carriers should have a certain nano-scale size to extravasate and accumulate in the tumor region. Third, the nanocarriers should be sufficiently internalized into HCC cells and escape from lysosomal degradation to release their cargos safely into the cytosol [30]. These collective challenges may, in part, account for the failure of in vivo delivery of gene therapies to HCC. Based on our understanding of these impediments, we designed a novel nanoparticle encapsulating anti-PD-1 antibody and MDK-siRNA to cope with them.

Toxicity studies revealed the biosafety of nanodrug as evidenced by hepato-renal functions and histopathological examinations thanks to its high selectivity with minimal effects on non-tumorous tissues [31]. In addition, lowered serum levels of AST, ALT, albumin and total bilirubin reflected the efficient inhibition of HCC progression with a subsequent positive prognosis. There was no evidence of systemic inflammatory or immune responses resulting from nanodrug administration, as evidenced by the normal levels of pro-inflammatory cytokines, which support the biosafety findings.

## Conclusions

In conclusion, we develop a novel tumor-targeted nanocarrier for the combined delivery of aPD-1 and MDK-siRNA. We confirmed the formed nanodrug (aPD-1-siRNA@NP) achieved a highly effective therapy and minimal side effects in vitro and in the orthotopic HCC model. Our efforts offered a nanomedicine strategy for tumor-targeted dual-drug delivery to gain remarkable efficacy in HCC treatment.

## Electronic supplementary material

Below is the link to the electronic supplementary material.

Supplementary Material 1

## Data Availability

The data that supports the findings of this study are available from the corresponding author,

## Abbreviations

HCC: Hepatocellular carcinoma
ICB: Immune checkpoint blockade
TME: Tumor microenvironment
TAMs: Tumor-associated macrophages
MDSCs: Myeloid-derived suppressor cells
PD-1: Programmed cell death receptor 1
PD-L1: Programmed cell death-ligand 1
MDK: Midkine
