# Supplementary material for: A novel pH-sensitive nanoparticles encapsulating anti-PD-1 antibody and MDK-siRNA overcome immune checkpoint blockade resistance in HCC via reshaping immunosuppressive TME
Source: J Exp Clin Cancer Res. 2025 May 16;44:148. doi: 10.1186/s13046-025-03396-6 (PMC12082952; doi:10.1186/s13046-025-03396-6)
Supplement: Supplementary file 1 — Supplementary Material 1 [file 13046_2025_3396_MOESM1_ESM.docx]

**A novel pH-sensitive nanoparticles encapsulating anti-PD-1 antibody and MDK-siRNA overcome immune checkpoint blockade resistance in HCC via reshaping immunosuppressive TME**

**Running title:** MDK-siRNA nanomedicine for HCC immunotherpay

**Supplementary Materials**

**METHODS**

1. **Synthesis and characterization of pH-sensitive copolymer and nanodrug**

The polymer was synthesized via multisteps and the successful synthesis of polymer was verified by proton nuclear magnetic resonance (1H NMR) and Fourier transform infrared (FTIR) analysis, including the following steps:

(1) Synthesis of Allyl-PEG-PBLA

(2) Synthesis of Allyl -PEG-PBLA-Ac

(3) Synthesis of HO-PEG-PBLA

(4) Synthesis of HO-PEG-PAsp(AzE)

(5) Synthesis of CDM-PEG-PAsp(AzE)

The polymer was synthesized via multisteps as shown in Figure S1 and the successful synthesis of polymer was verified by proton nuclear magnetic resonance (1H NMR) and Fourier transform infrared (FTIR) analysis. α-Allyl poly(ethylene glycol)-b-poly (β-benzyl L-aspar-tate), namely, Allyl-PEG-PBLA, was first synthesized by a ring opening polymerization of BLA-NCA using the Allyl-PEG-NH2 as an initiator. As shown in Figure S2-S6, the characteristic peaks of benzyl-aspartate units (7.28, 5.03, 4.60, and 2.51~2.92 ppm) and methylene of initiator (3.52 ppm) were clearly shown, indicating the successful synthesis. The average degree of polymerization for PBLA was calculated to be 32, by comparing the characteristic chemical shift integral of the benzyl group (7.28 ppm) and the methylene group (3.52 ppm) in the 1H NMR spectra. Then, 2-mercaptoethanol was reacted with the allyl group by Michael addition reaction, showing the disappearance of characteristic peaks for allyl double bond (5.54-5.60 ppm) meanwhile the appearance of characteristic peak for 2-mercaptoethanol (3.33 ppm) by 1H NMR analysis (Figure S4). Subsequently, HO-PEG-PAsp(AzE) was synthesized by aminolysis of HO-PEG-PBLA(poly-beta-benzyl-L-aspartate) with 2-(azepan-1-yl)ethanamine (AzE). After the aminolysis, the characteristic peaks of benzyl groups (7.28 ppm) disappeared whereas the characteristic peaks of methylene groups of AzE appeared (1.66, 3.09 ppm) (Figure S5). The completed aminolysis was also verified by FTIR analysis (Figure S7), showing the disappearance of characteristic vibration absorption peaks for benzyl ester in PBLA at 1730 cm-1 (νC=O, ester), 745 cm-1 and 696 cm-1 (δPh-H). Finally, 2-propionic acid-3-methyl maleic anhydride (CDM) was conjugated to the polymer, which resulted in a characteristic peak (2.13 ppm) in the 1H NMR spectra of CDM-PEG-PAsp(AzE) (Figure S6).

The pH-sensitive copolymer was self-assembled into a micelle with MDK-siRNA encapsulated in the hydrophobic core (siMDK@NP). Then aPD-1 was conjugated to the surface of as-prepared micelle to obtain final nanodrug (aPD-1-siMDK@NP). The loading content of MDK-siRNA and aPD-1 were 6.4±0.4% and 3.5±0.3%, as detected by UV-vis and ELISA, respectively. Measurement with DLS analysis showed that the particle sizes of aPD-1-siMDK@NP was 120.5±8.7 nm and 102.1±7.4 at pH 7.4 and 6.5 (Figure S12) . The decreased particles size of nanodrug at pH 6.5 might be attributed to the release of aPD-1. As demonstrated by TEM observation, aPD-1-siMDK@NP exhibited a spherical morphology around 100 nm at pH 7.4 (Figure S10), which was in line with the results of DLS detection. Additionally, a darker aPD-1 layer on the nanodrug surface was clearly observed, since the antibody is much easier to be stained with uranyl acetate. When the pH value changed to 6.5, the nanoparticles became smaller; meanwhile, the aPD-1 layer disappeared (Figure S11), implying the antibody was shed from nanodrug.

For preparation of antibody-decorated nanodrug, the anti-PD-1 antibody was added into the aqueous solution containing tris(2-carboxyethyl)phosphine hydrochloride (TCEP), shook for 5 min and left static for half an hour. Then, the antibody solution was added to the siMDK@NP solution and incubated at 4 C overnight. To remove TCEP, the solution was concentrated and washed three times with PBS (pH 7.4, 0.02 M) via a MILLIPORE Centrifugal Filter Device (MWCO: 100 kDa). After being filtered through a syringe filter (pore size: 0.22 μm), the nanodrug aPD-1-siMDK@NP was prepared. More pieces of evidence were obtained from the in vitro aPD-1 release assay, finding nearly 60% aPD-1 was released from nanodrug at pH 6.5 within 4 hours (Figure S14). The results demonstrated that the antibody could be efficiently released responded to TME acidity, which was essential for aPD-1 to activate the cytotoxic T cells. Moreover, the aPD-1 release induced a potential reversal of nanodrug from −9.5±1.3 mV to+3.3 ± 0.6 mV, owing to the shedding of negatively charged antibody and the protonation of AzE groups, which might facilitate the endocytosis of nanodrug by TAMs and MDSCs.

**2. Biosafety of nanodrug**

Acute and chronic toxicity studies were conducted to confirm the biosafety of nanodrug. In the acute toxicity study, HCC-bearing mice were injected intravenously with nanodrug at an siRNA dose of 3 mg/kg. At 24 h post administration, serum samples were collected, centrifuged for 30 min (1200 g, 4 °C) and the plasma was separated. Various markers associated with hepato-renal functions were assayed, including alanine aminotransferase (ALT), aspartate aminotransferase (AST), albumin and total bilirubin (TBIL). The gene expression of pro-inflammatory cytokines IL-6, TNF-α and IFN-γ was assessed in the tumor tissues 24 h post administration.

In the chronic toxicity study, serum was collected from mice at the end of the repeated treatment by nanodrug according to the same schedule. Hepato-renal function markers were assayed in plasma and normalized to HEPES buffer-treated mice. A histo-pathological examination for the liver of mice was also carried out after the chronic treatment to detect any potential injury.

**Supplementary figures**


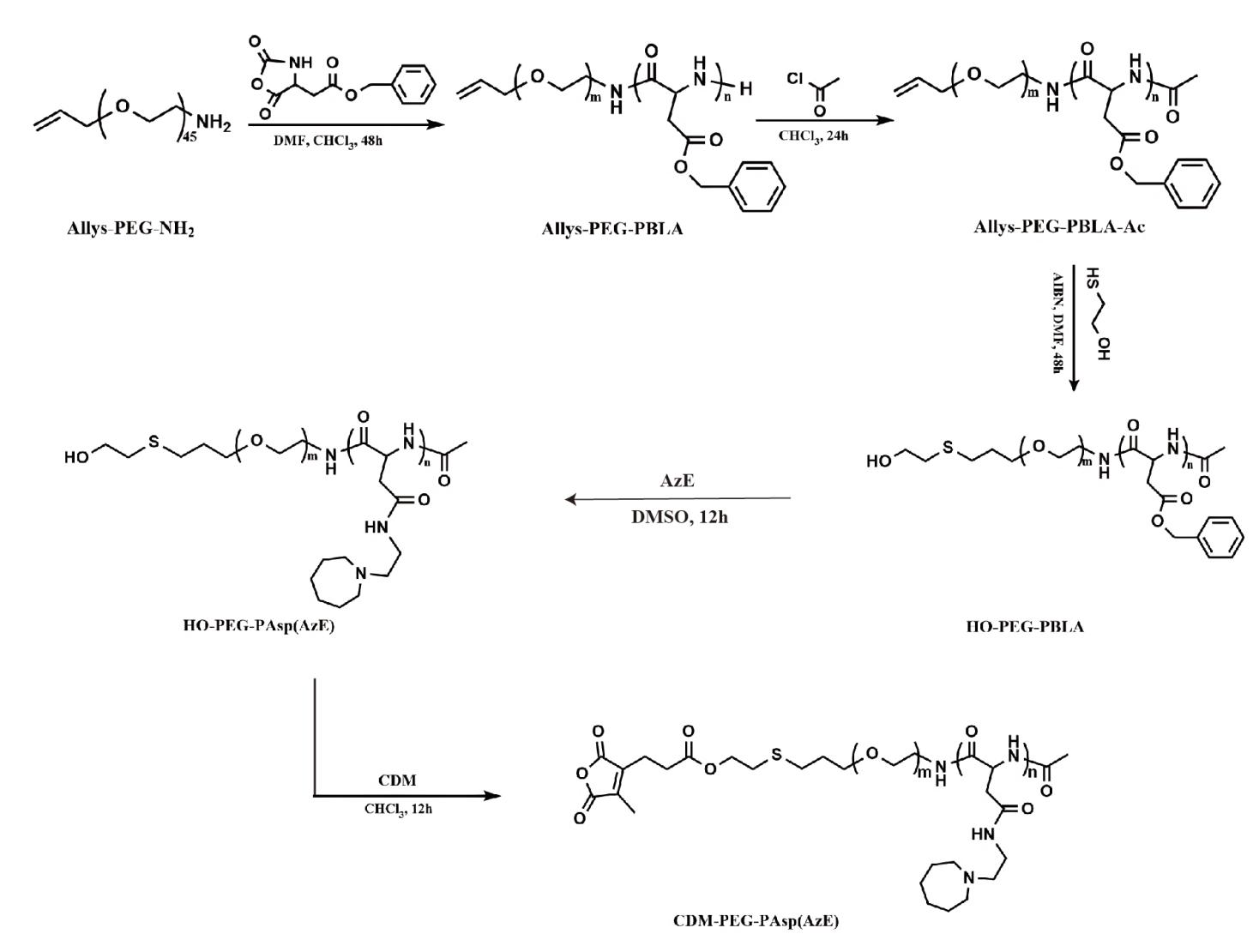


**Fig. S1. Synthesis of CDM-PEG-PAsp(AzE)**


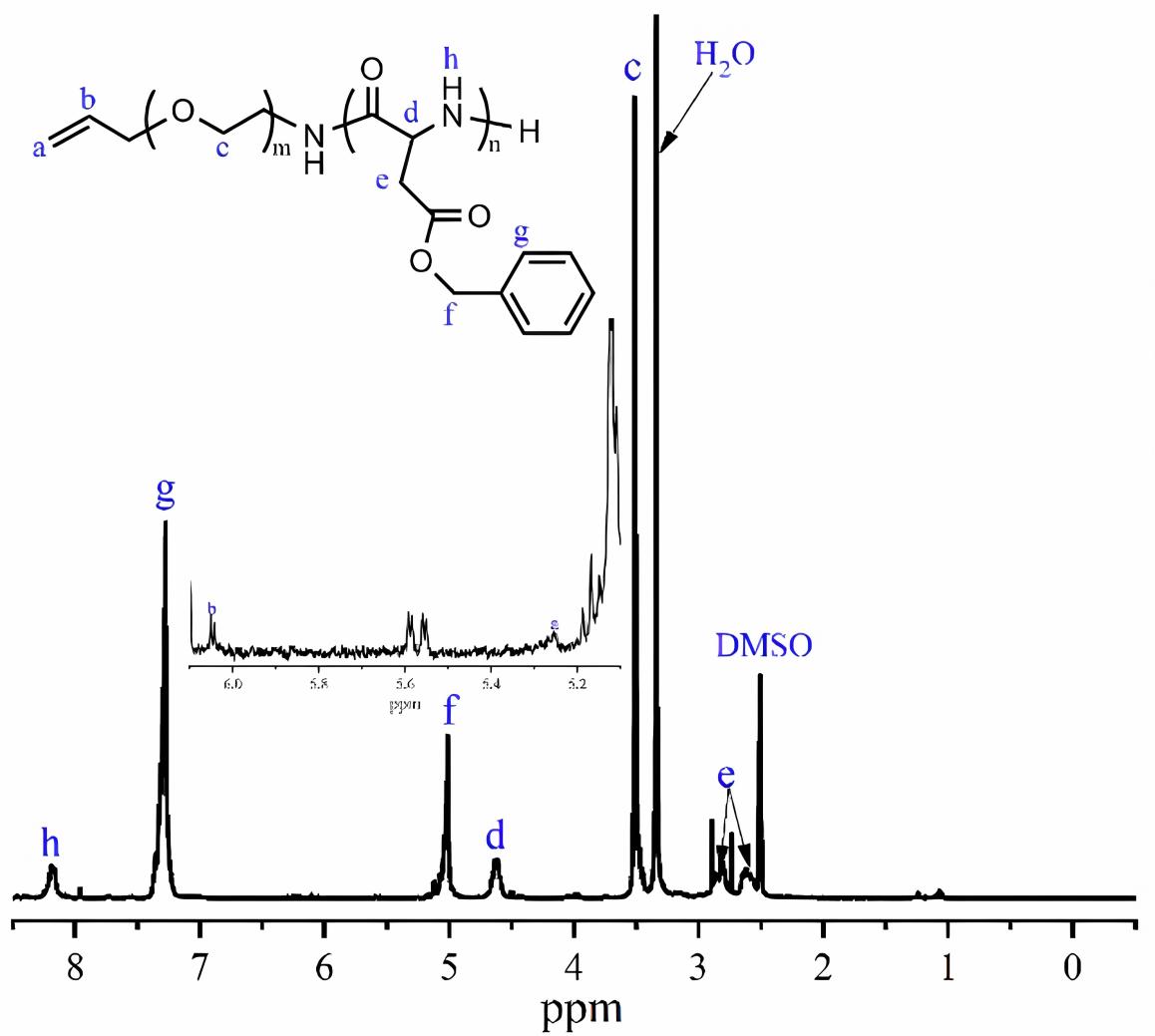


**Fig. S2. NMR Spectra of Allyl-PEG-PBLA (1H NMR) in DMSO-d_6_.**


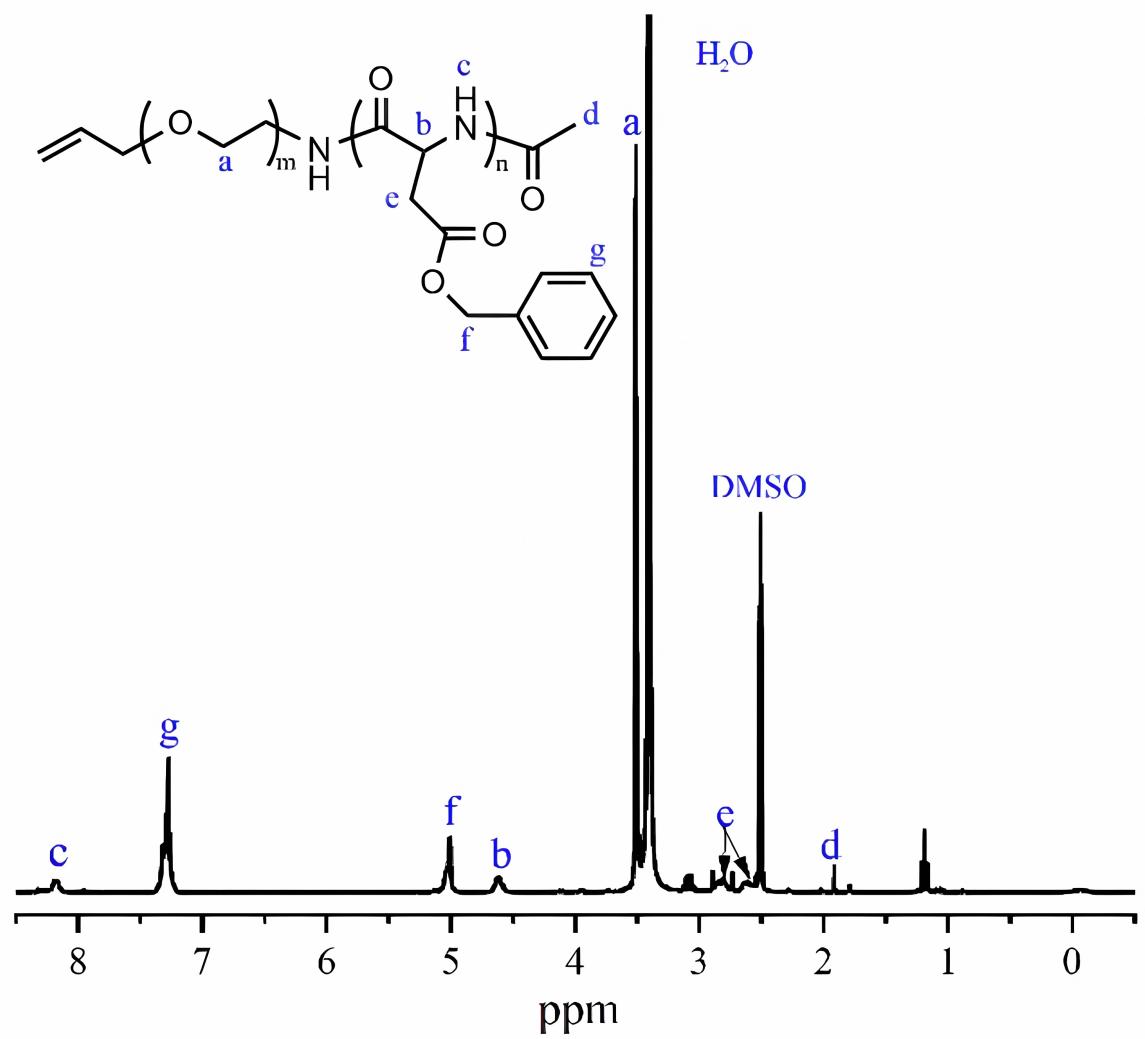


**Fig. S3. NMR Spectra of Allyl -PEG-PBLA-Ac (1H NMR) in DMSO-d_6_.**


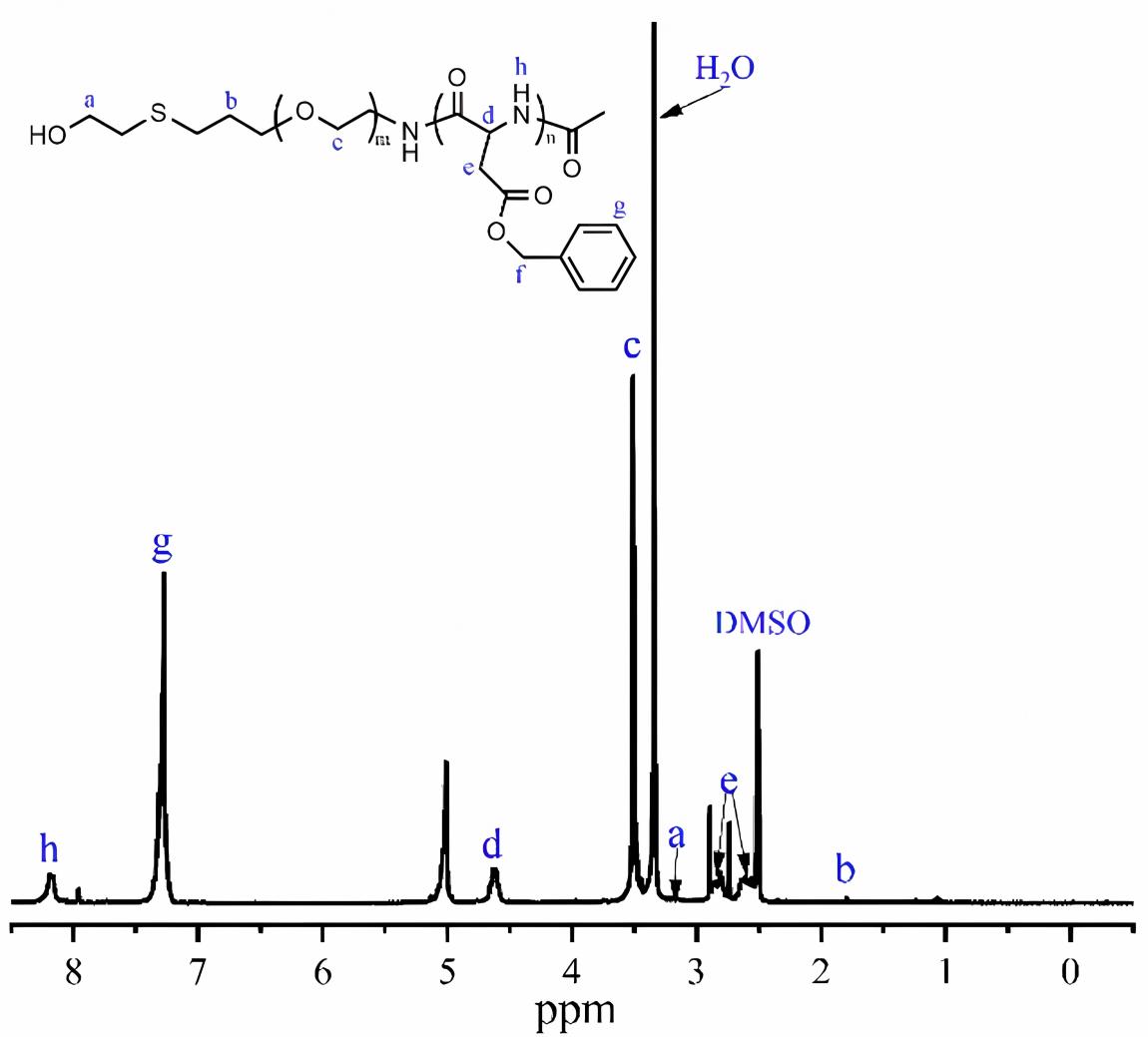


**Fig. S4. NMR Spectra of HO-PEG-PBLA (1H NMR) in DMSO-d_6_.**


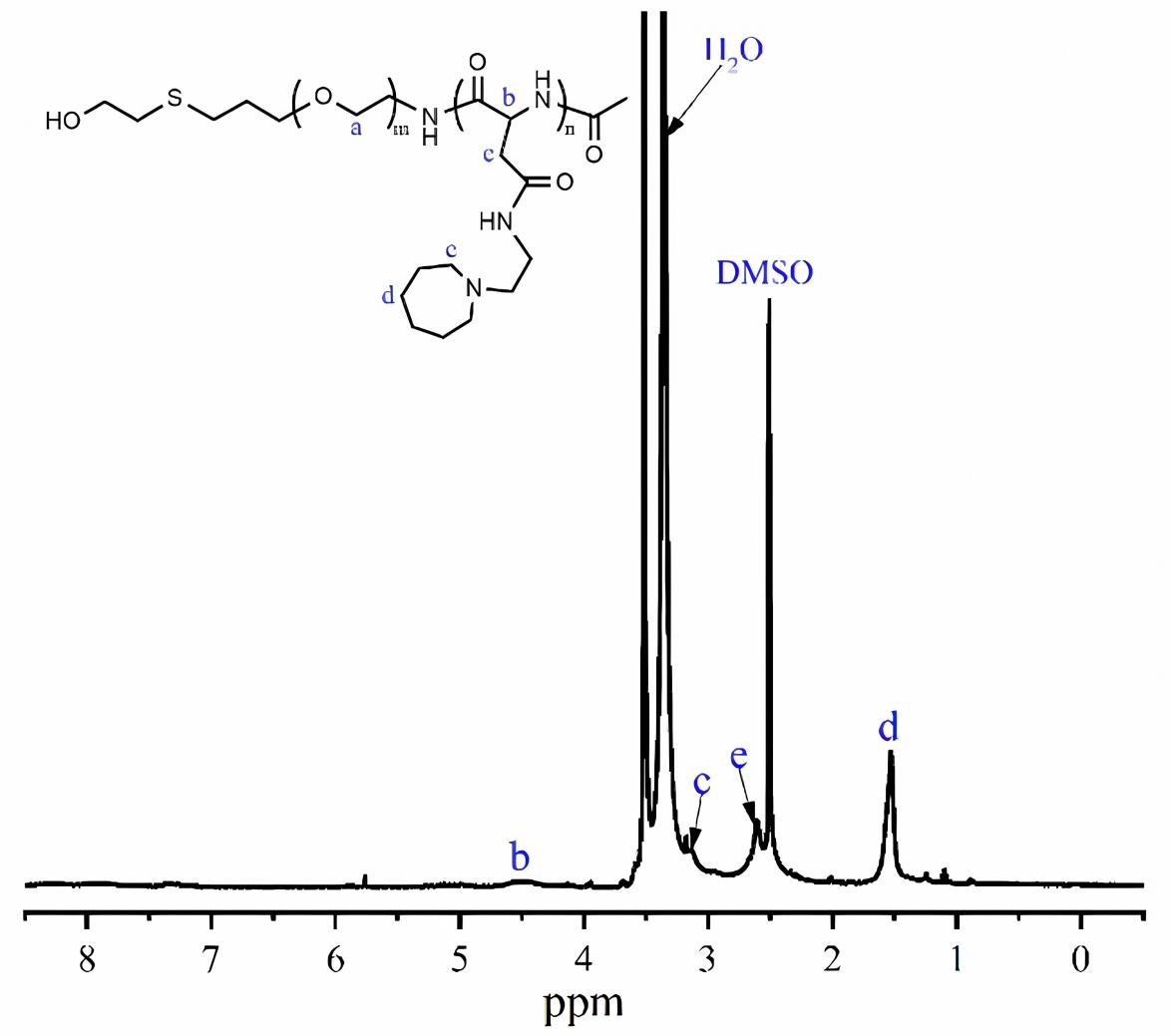


**Fig. S5. NMR Spectra of HO-PEG-PAsp(AzE) (1H NMR) in DMSO-d_6_.**


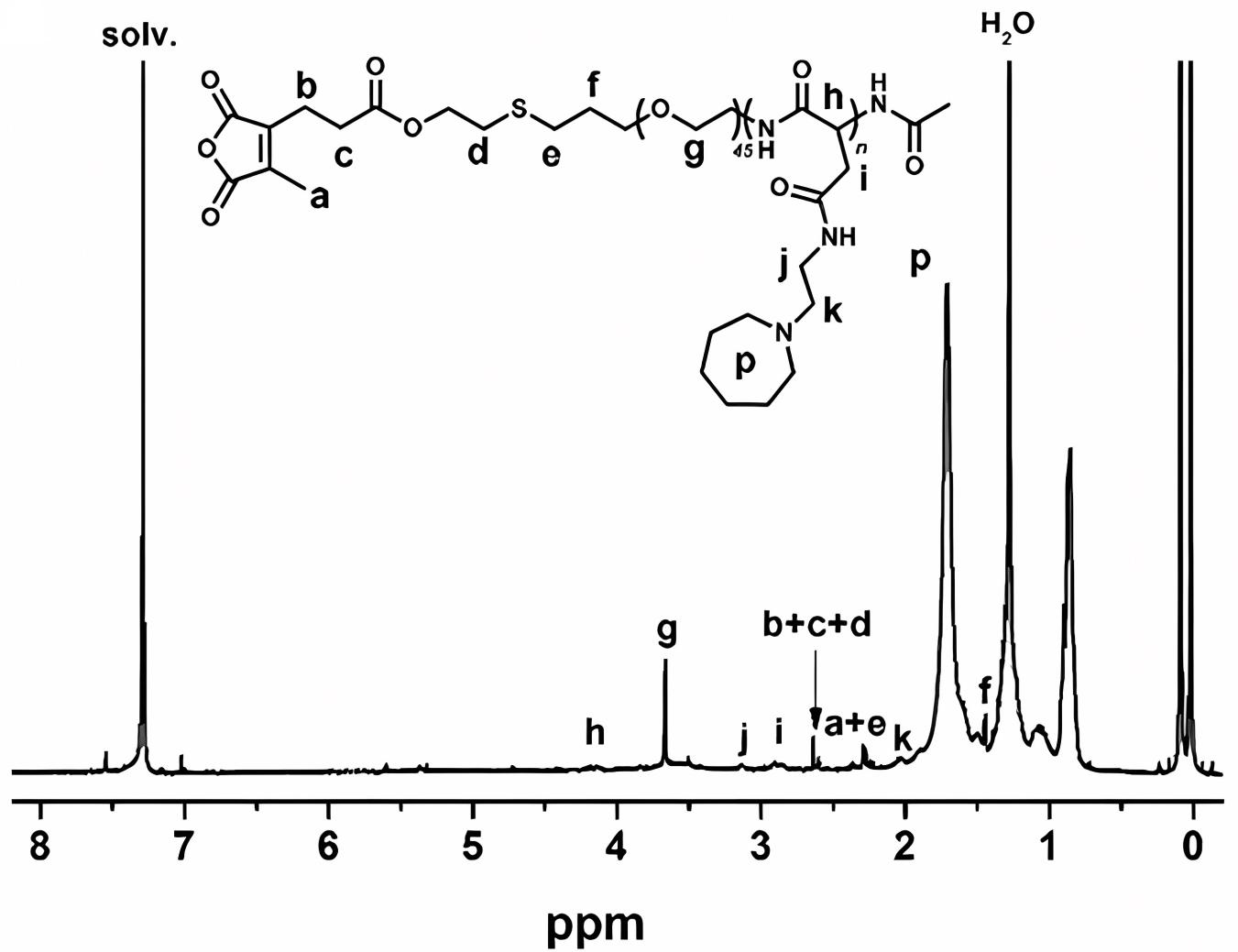


**Fig. S6. NMR Spectra of CDM-PEG-PAsp(AzE) (1H NMR) in DMSO-d_6_.**


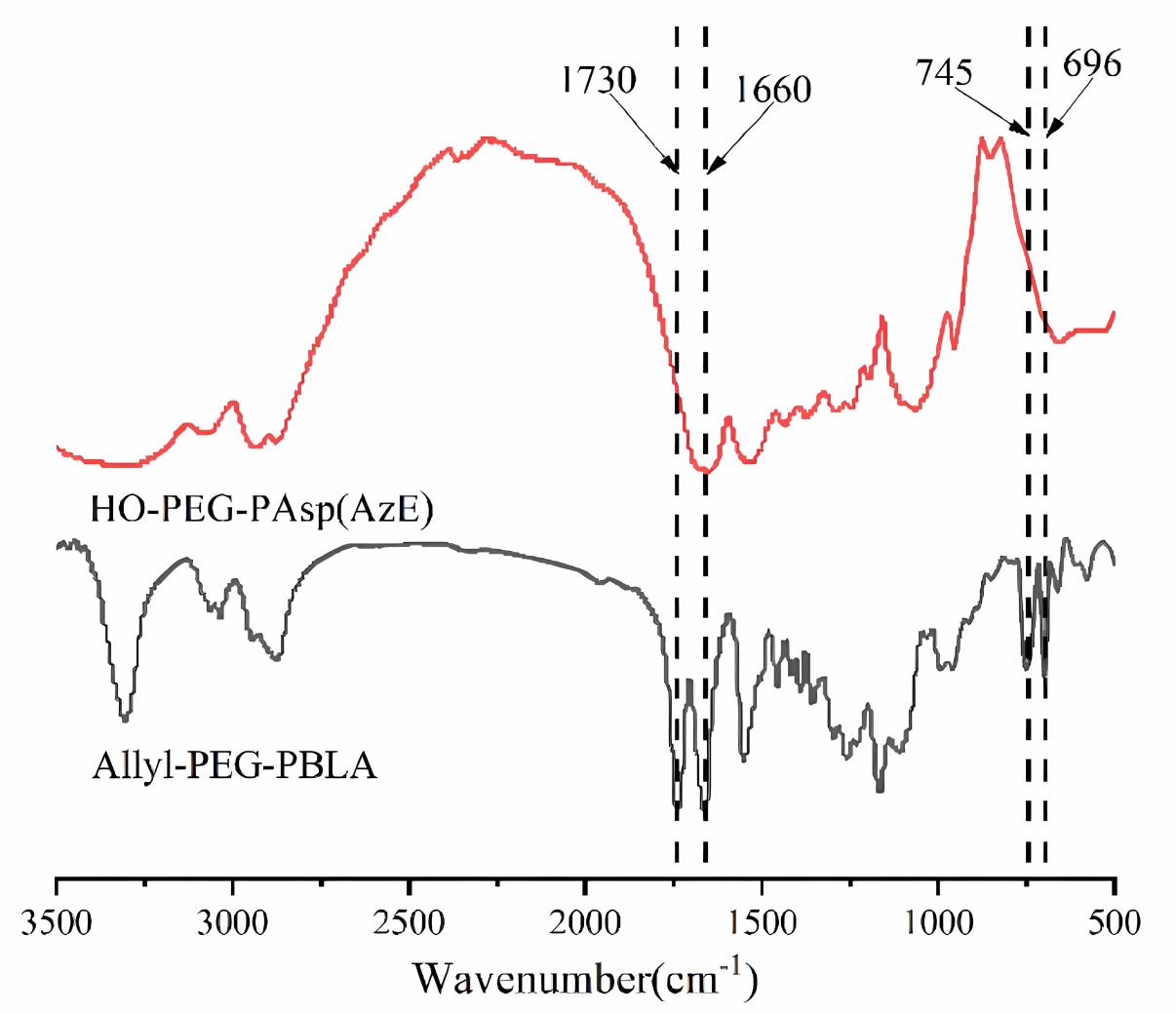


**Fig. S7. Fourier transform infrared (FTIR) analysis of Allyl-PEG-PBLA and HO-PEG-PAsp(AzE)**


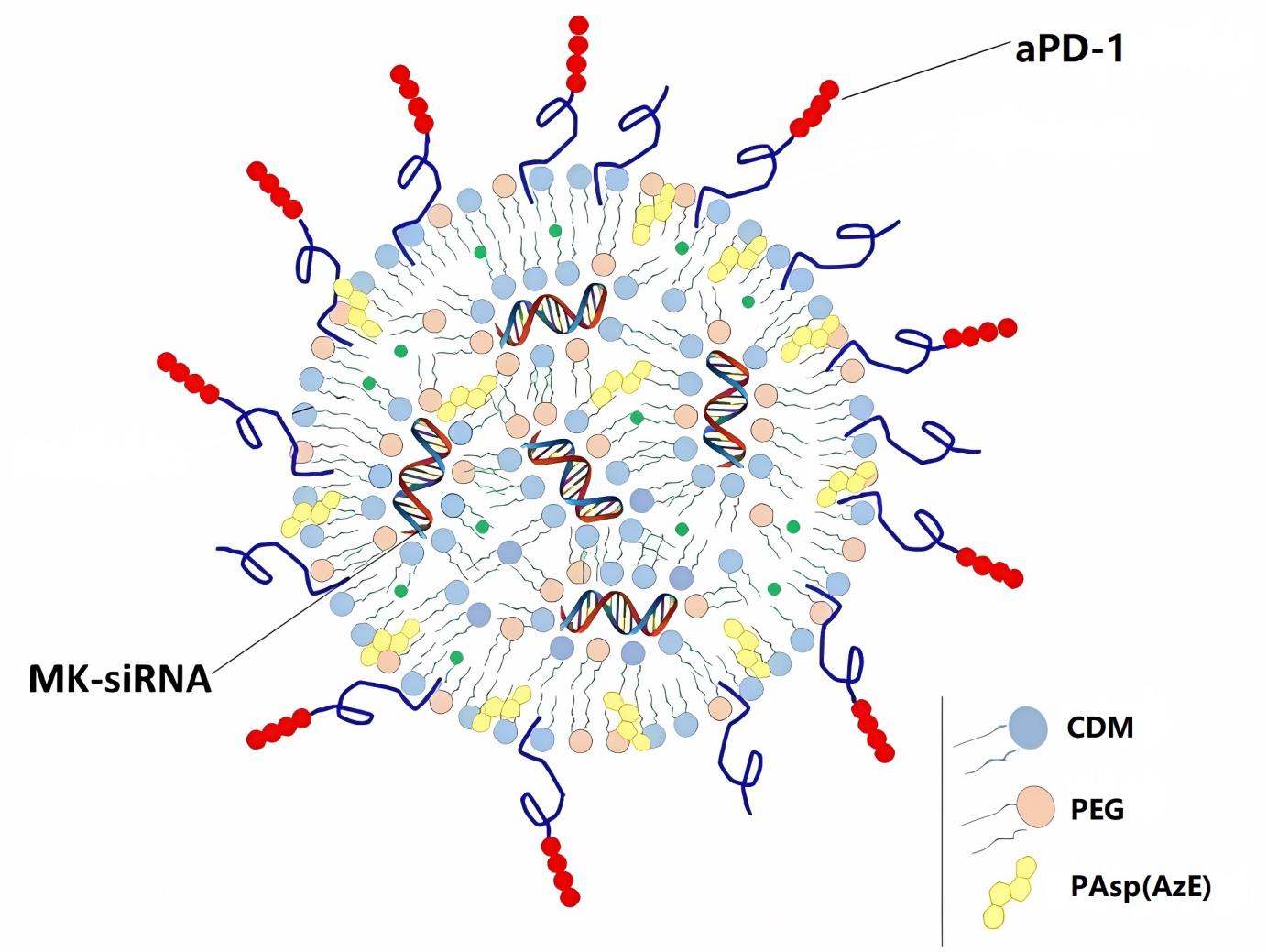


**Fig. S8. Composition diagram of nanomedicine (aPD-1-siMDK@NP)**


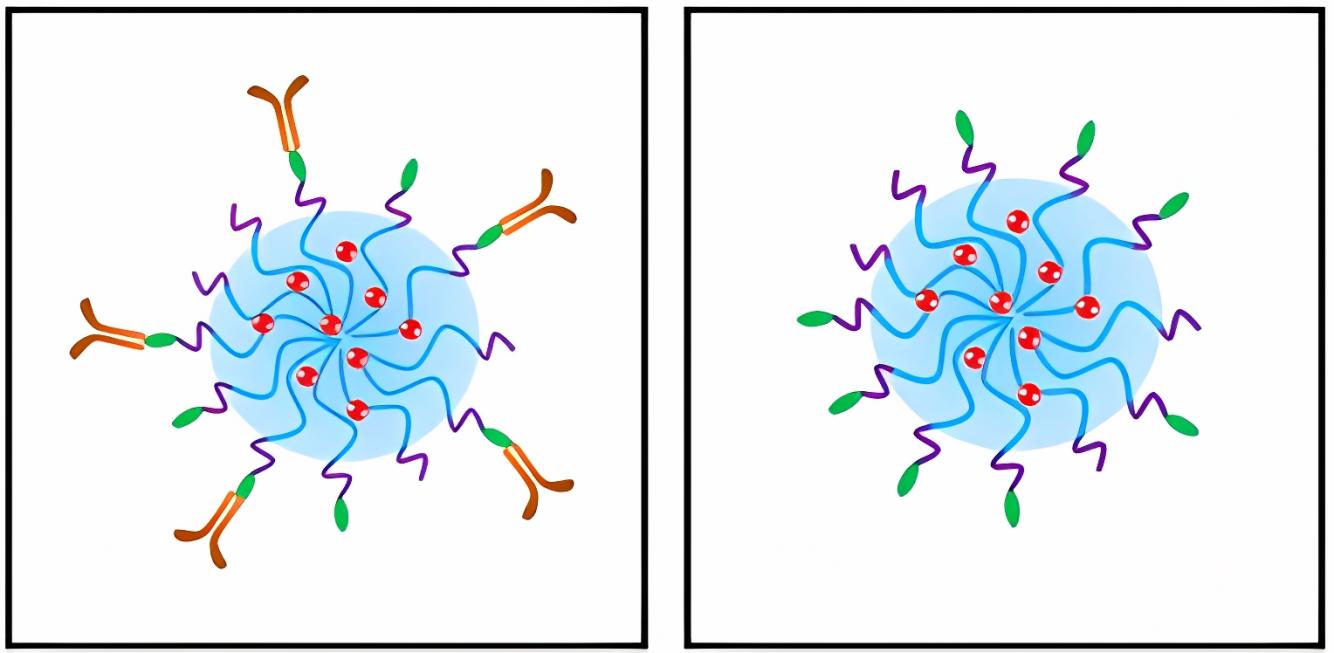


**Fig. S9. A TME acid cleavable linkage was introduced between aPD-1 and nanomicelle to endow a rapid shedding of antibody in TME, releasing aPD-1 for ICB and leaving residual MDK-siRNA-encapsulated nanomicelle. (left) pH 7.4; (right) pH 6.5**


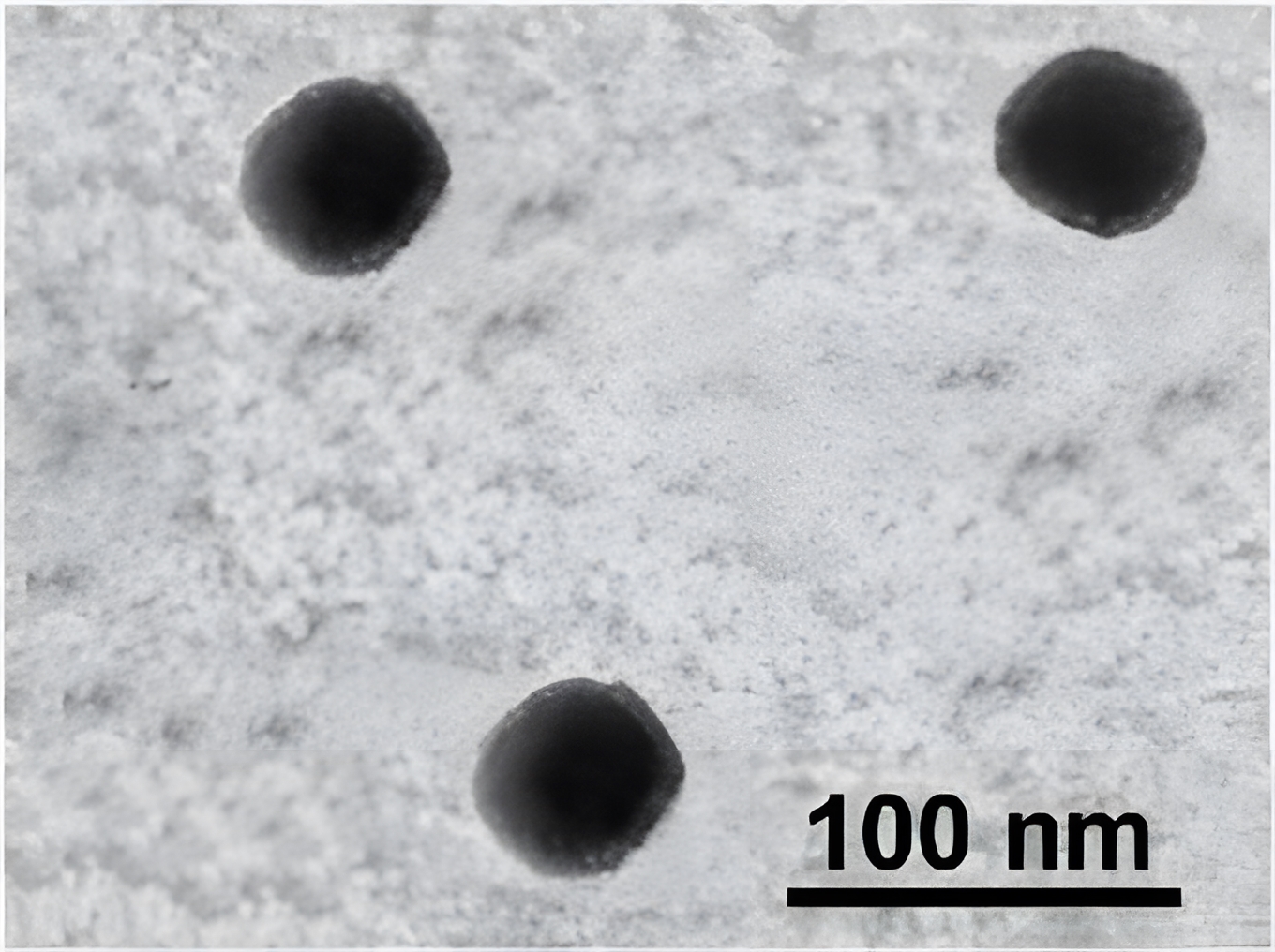


**Fig. S10. Transmission electron microscopy (TEM) image of nanomedicine (aPD-1-siMDK@NP)**


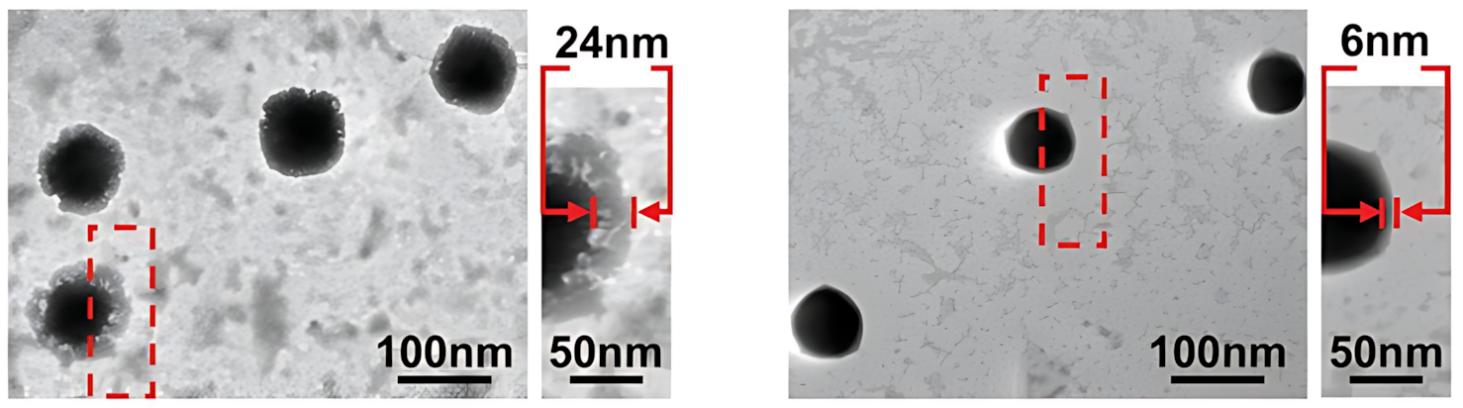


**Fig. S11. Transmission electron microscopy (TEM) image of nanomedicine (aPD-1-siMDK@NP) under (left) pH 7.4 or (right) pH 6.5**


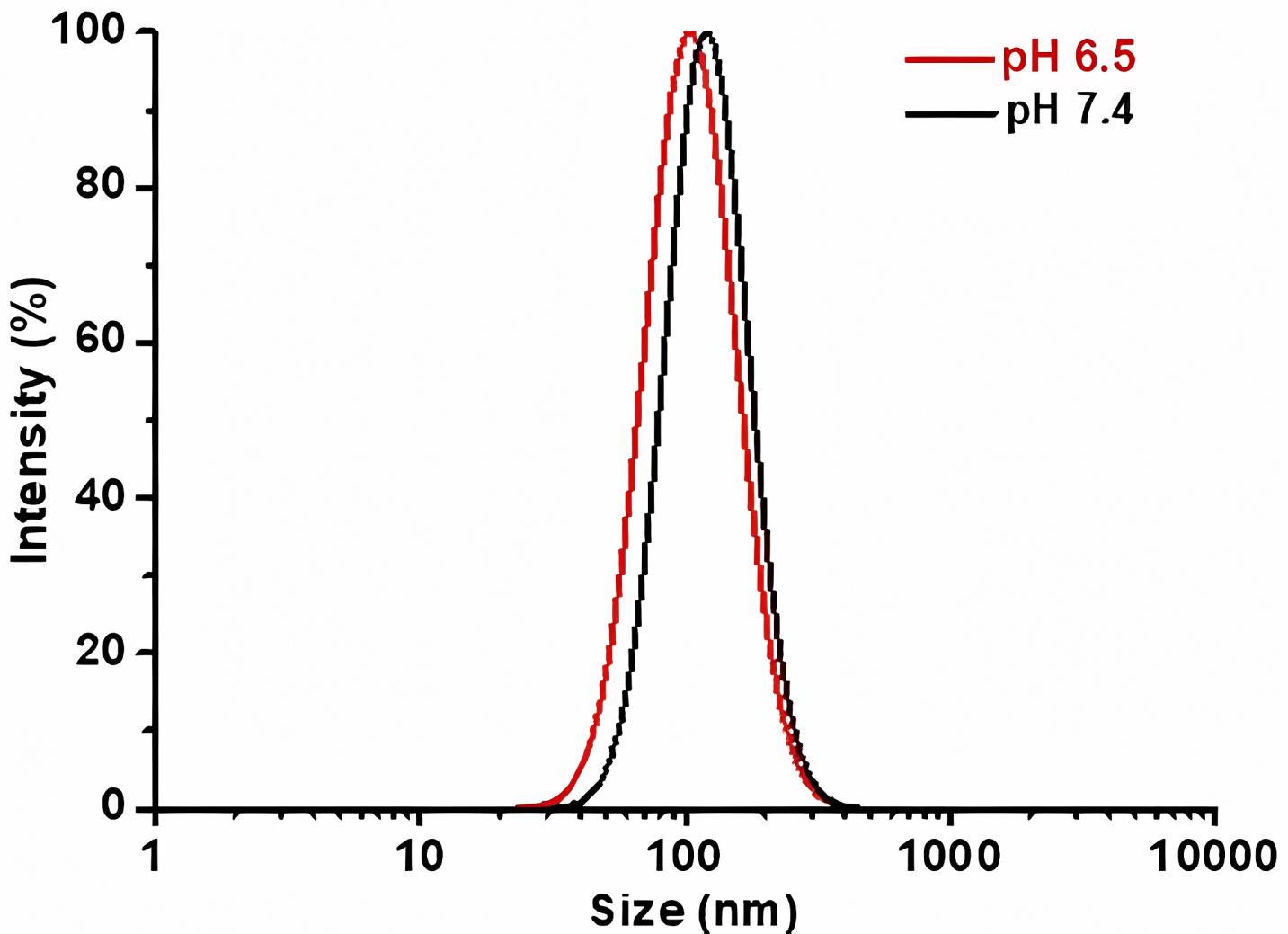


**Fig. S12. The diameters of nanomedicine (aPD-1-siMDK@NP) at pH 7.4 and pH 6.5 were measured by dynamic light scattering (DLS)**


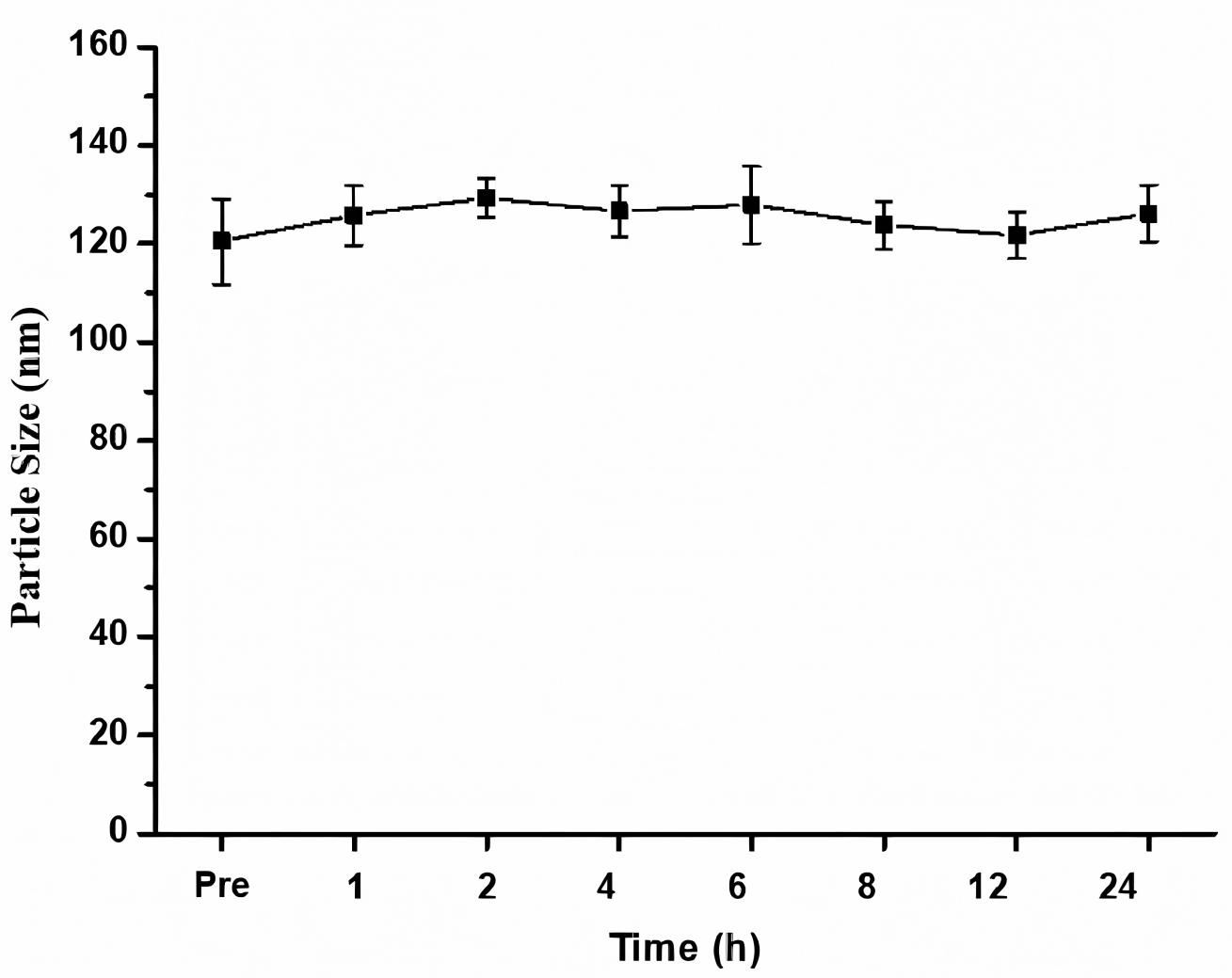


**Fig. S12. Serum stability of nanomedicine (aPD-1-siMDK@NP)**


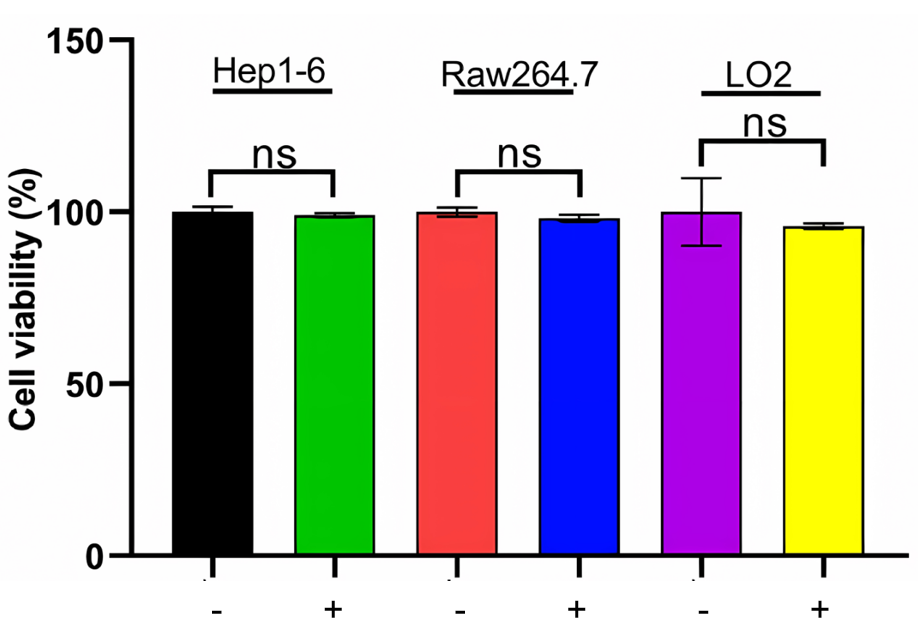


**Fig. S13. Cytotoxicity of nanomedicine (aPD-1-siMDK@NP)**


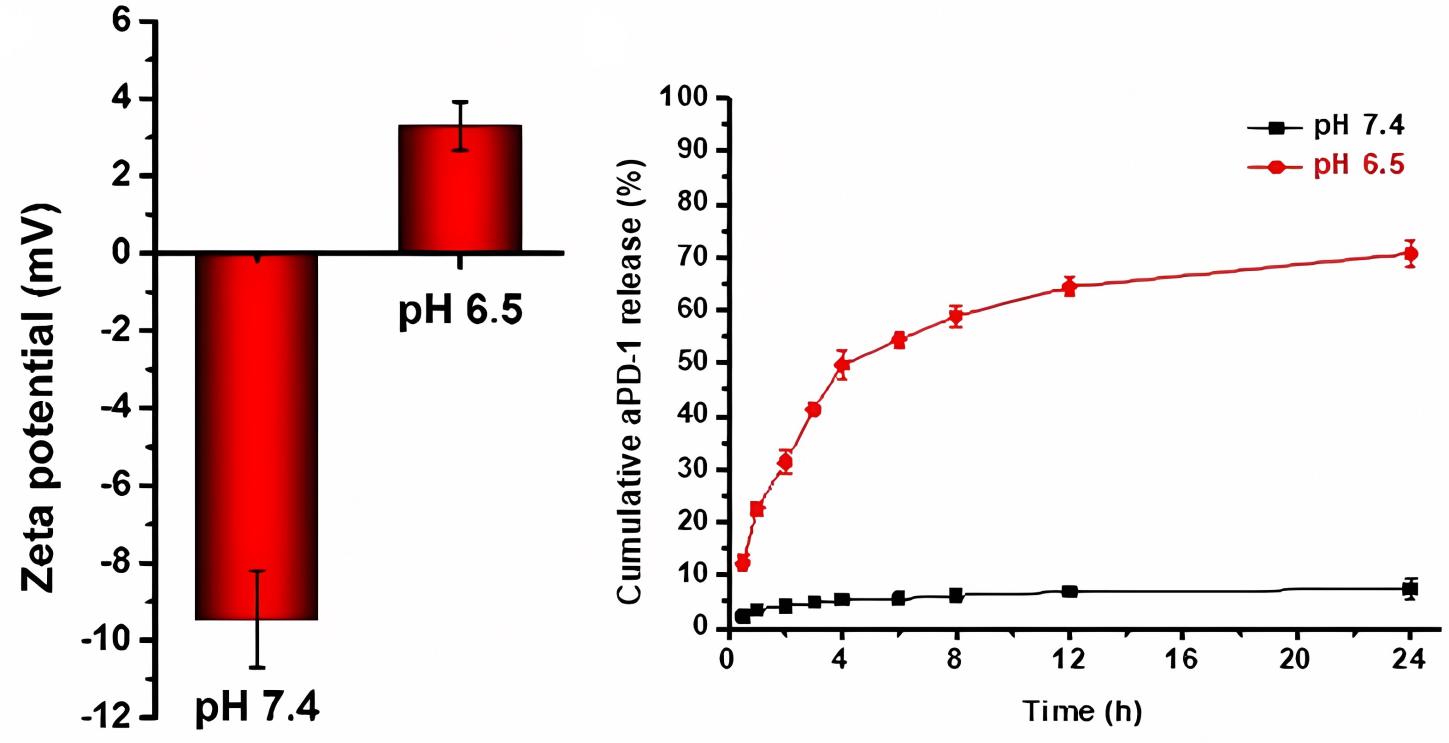


**Fig. S14. aPD-1 release assay in vitro**


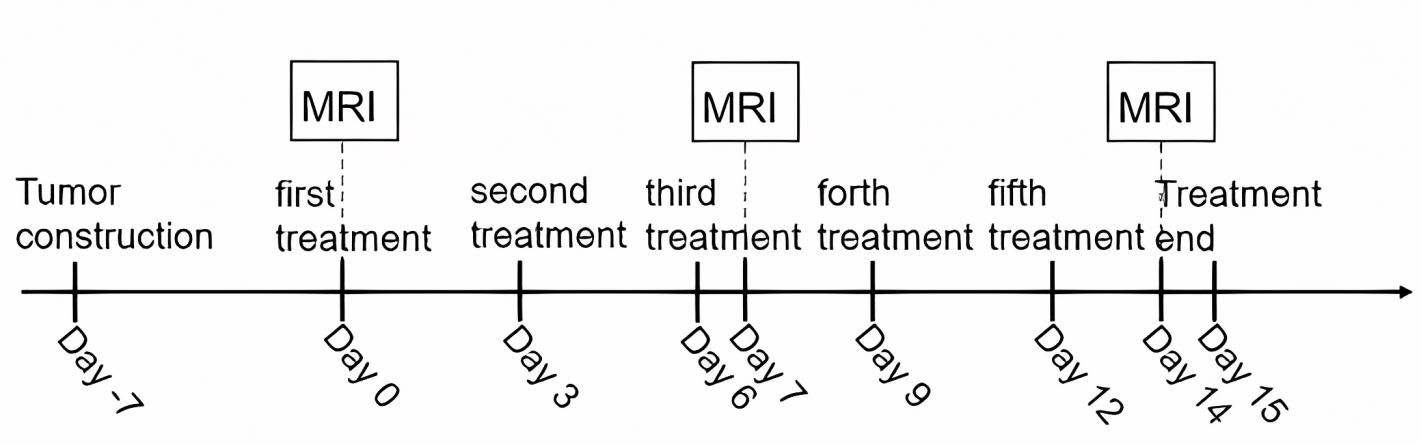


**Fig. S15. Nanomedicine treatment of HCC mice in situ**


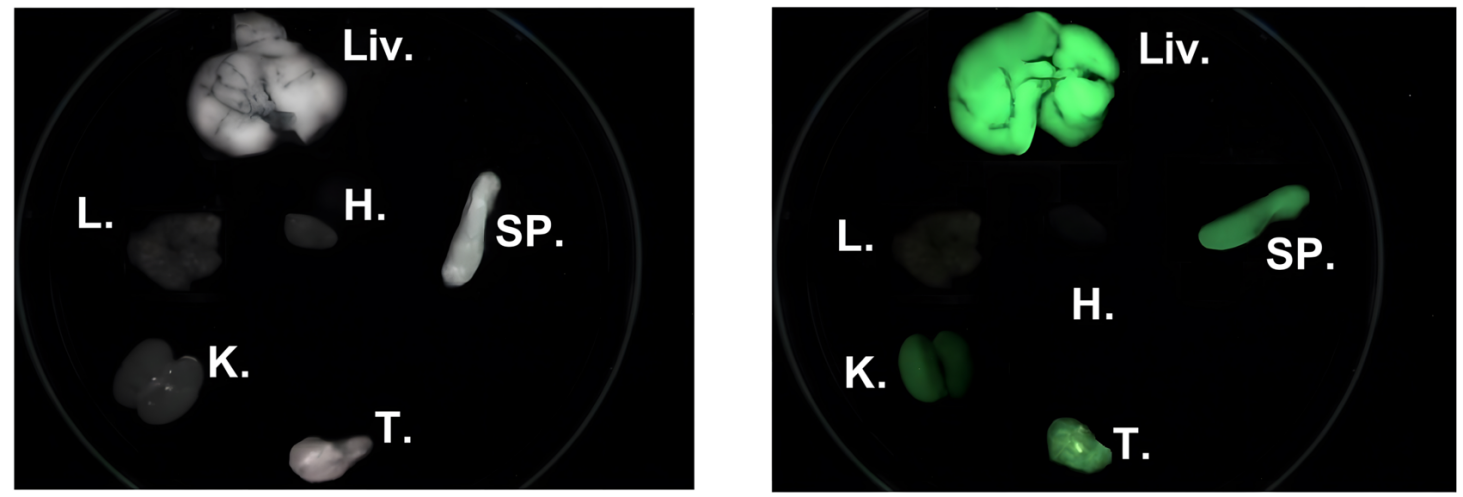


**Fig. S16. Biodistribution of nanodrugy. (left) Did-labeled nanomedicine distribution in vivo (24 hours after injection); (right) In vivo distribution of nanomedicine enveloping Alexa Fluor 488 target siRNA (24 hours after injection).**


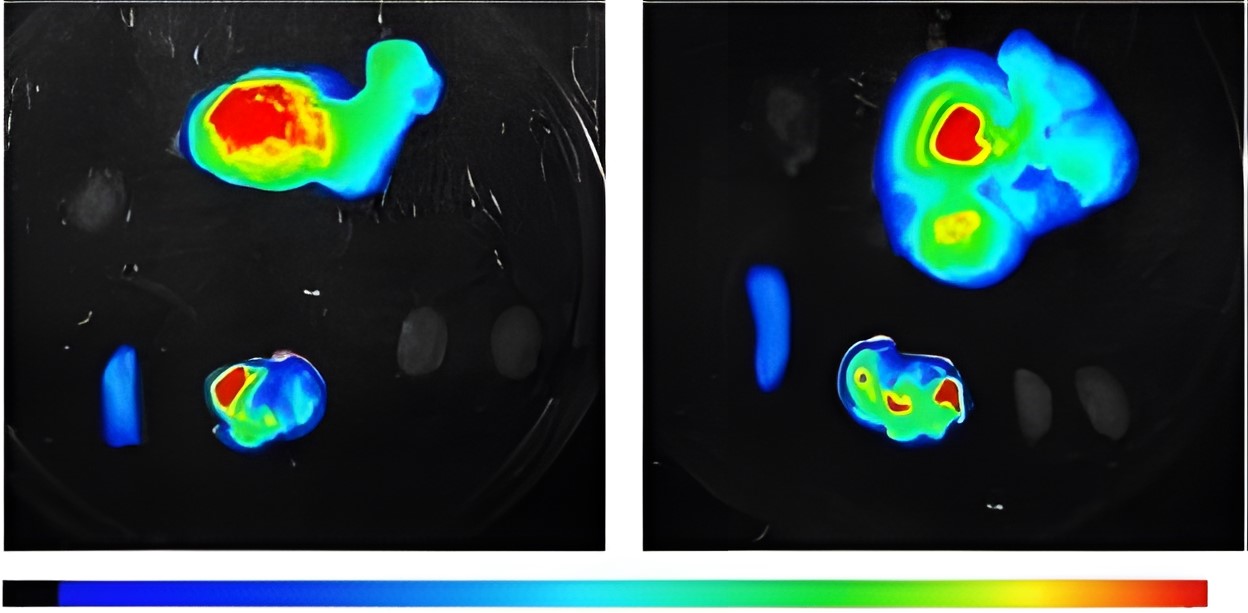


**Fig. S17. In vivo fluorescence imaging confirms the tumor targeted drug delivery ability of nanomedicine. (left) Did-labeled nanomedicine distribution in vivo (24 hours after injection); (right) In vivo distribution of nanomedicine enveloping Alexa Fluor 488 target siRNA (24 hours after injection).**


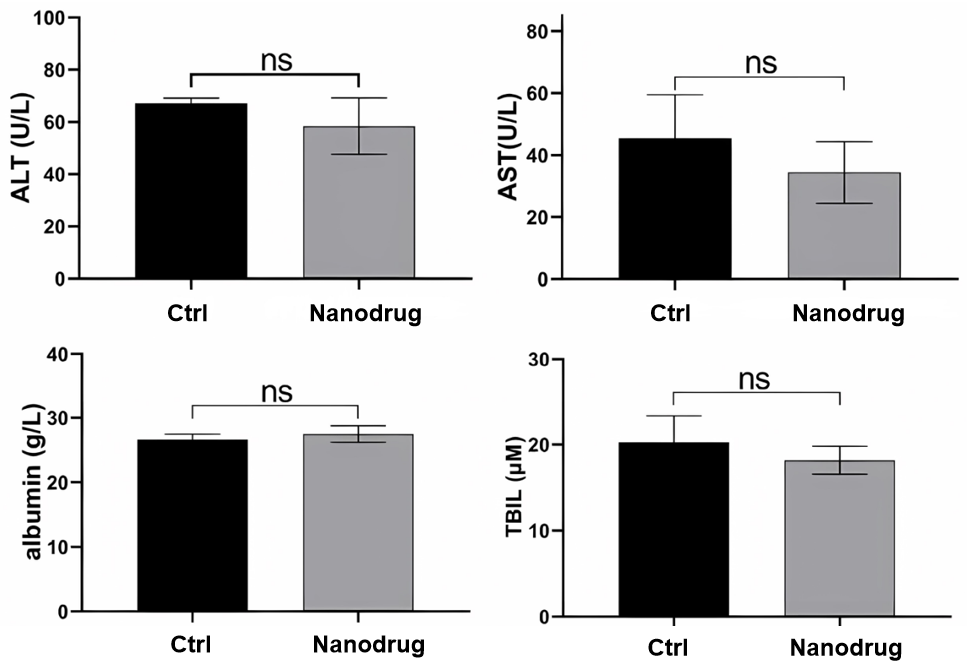


**Fig. S18. Biosafety of nanomedicine. Alanine aminotransferase (ALT); Aspartate aminotransferase (AST); Albumin Total bilirubin (TBIL).**


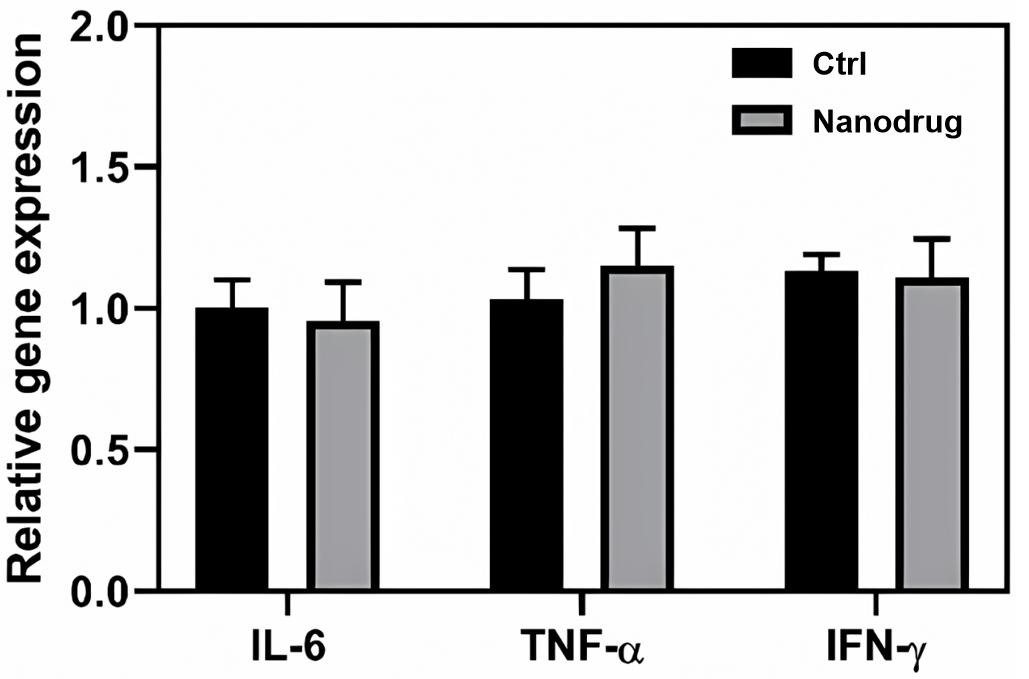


**Fig. S19. Biosafety of nanomedicine.**


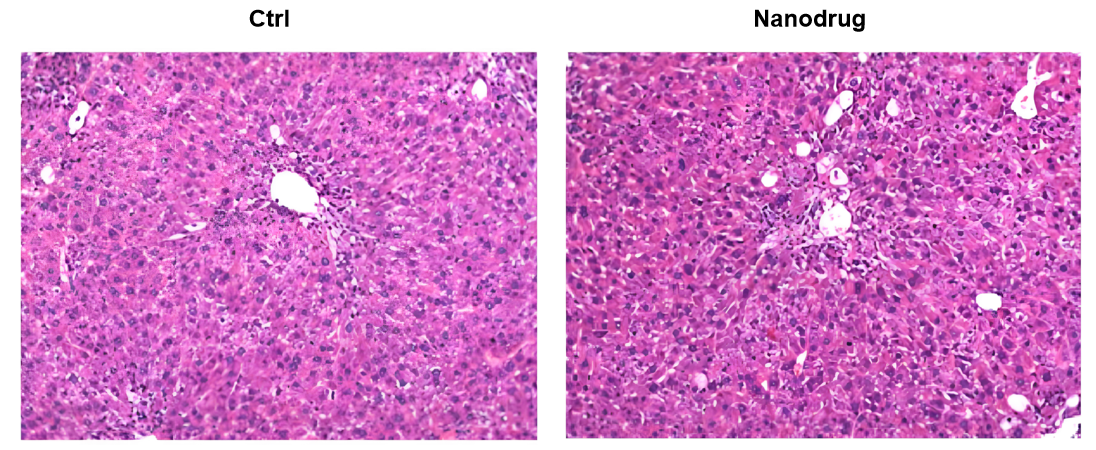


**Fig. S20. Biosafety of nanomedicine.**


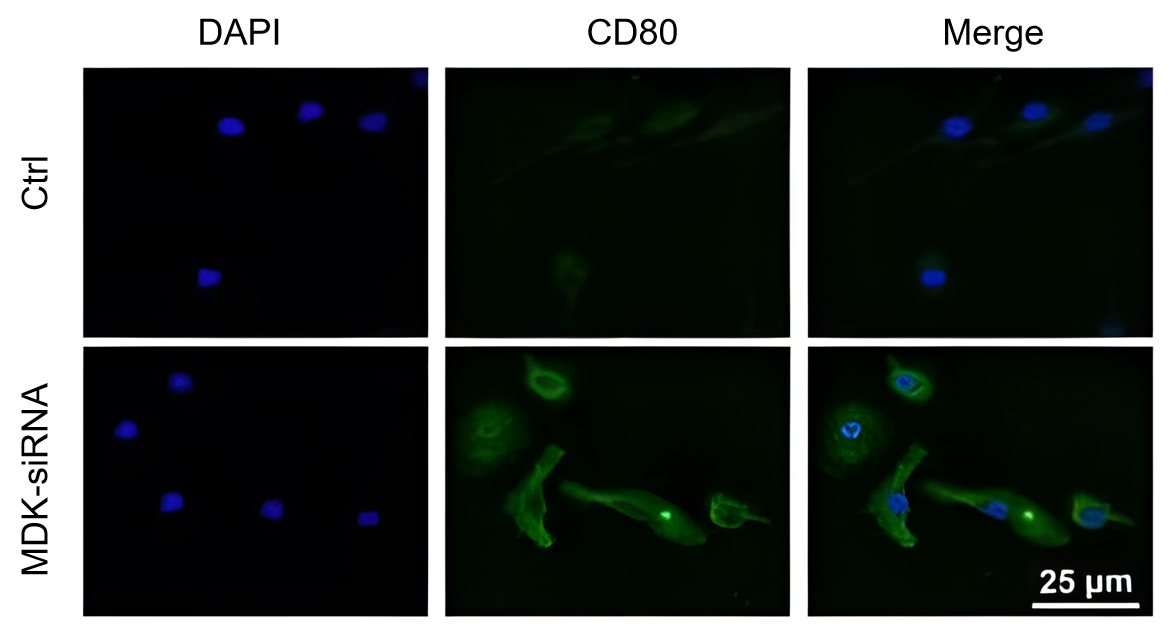


**Fig. S21. MDK-siRNA promotes M1 polarization in macrophages (CD80 as a marker)**


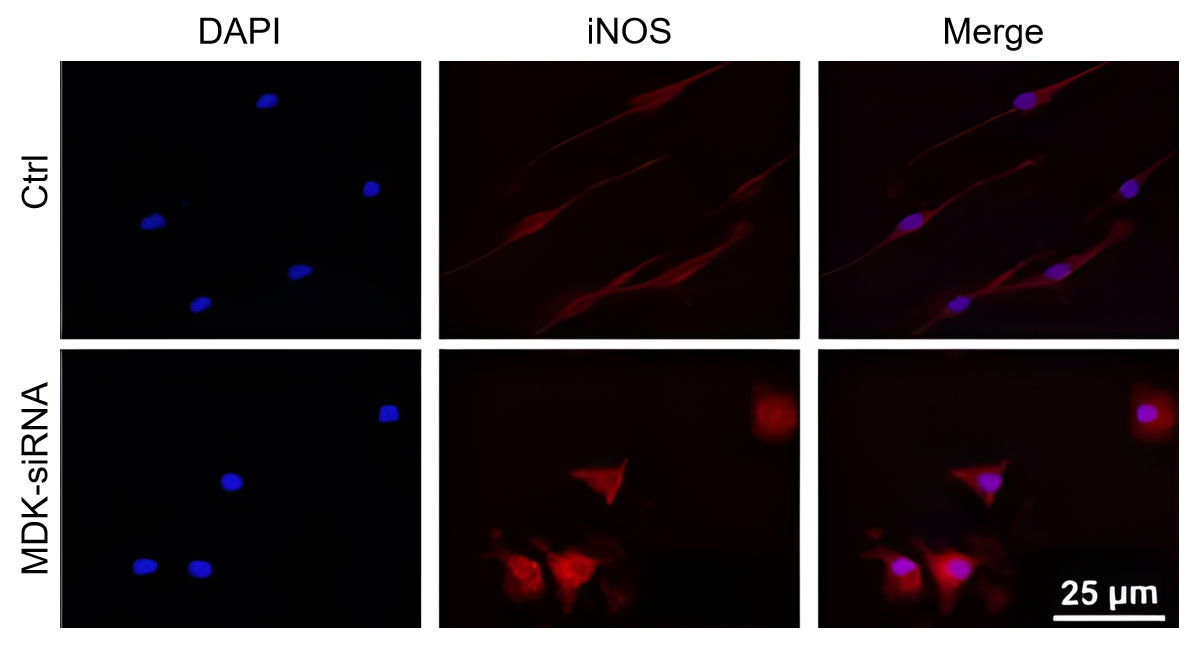


**Fig. S22. MDK-siRNA promotes M1 polarization in macrophages (iNOS as a marker)**


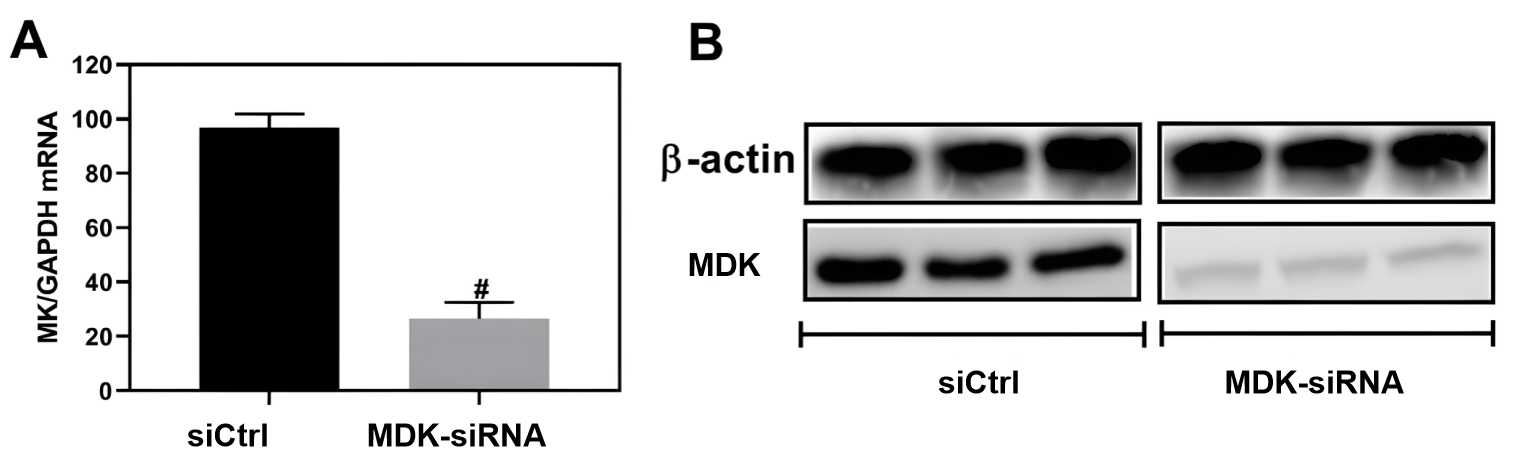


**Fig. S23. MDK-siRNA decreased MDK expression in MDSC**


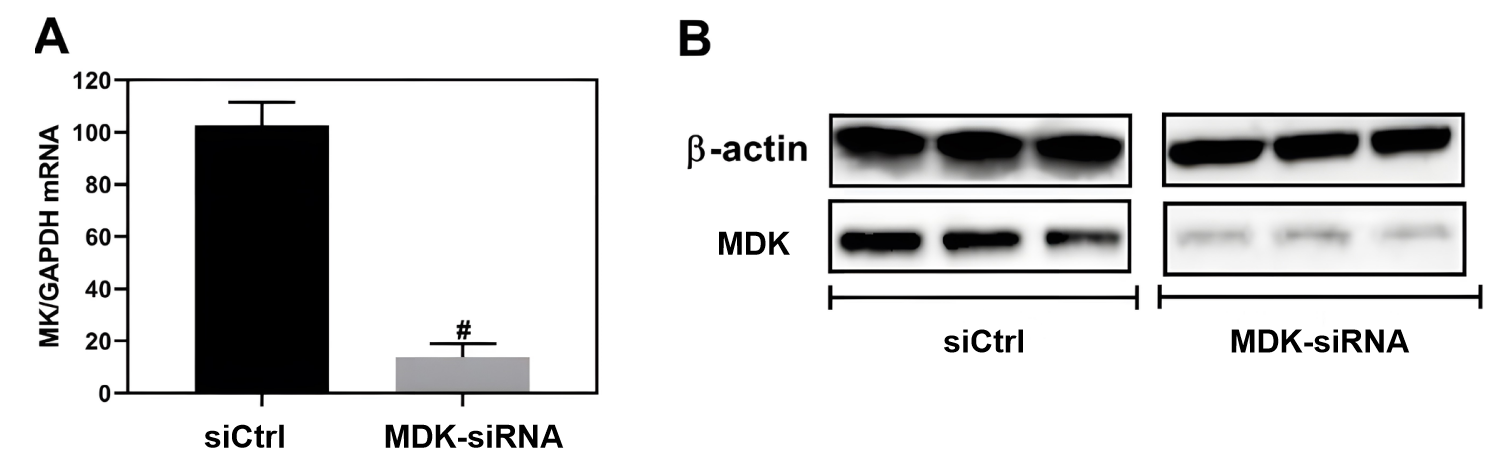


**Fig. S24. MDK-siRNA decreased MDK expression in M2-TAM**

**
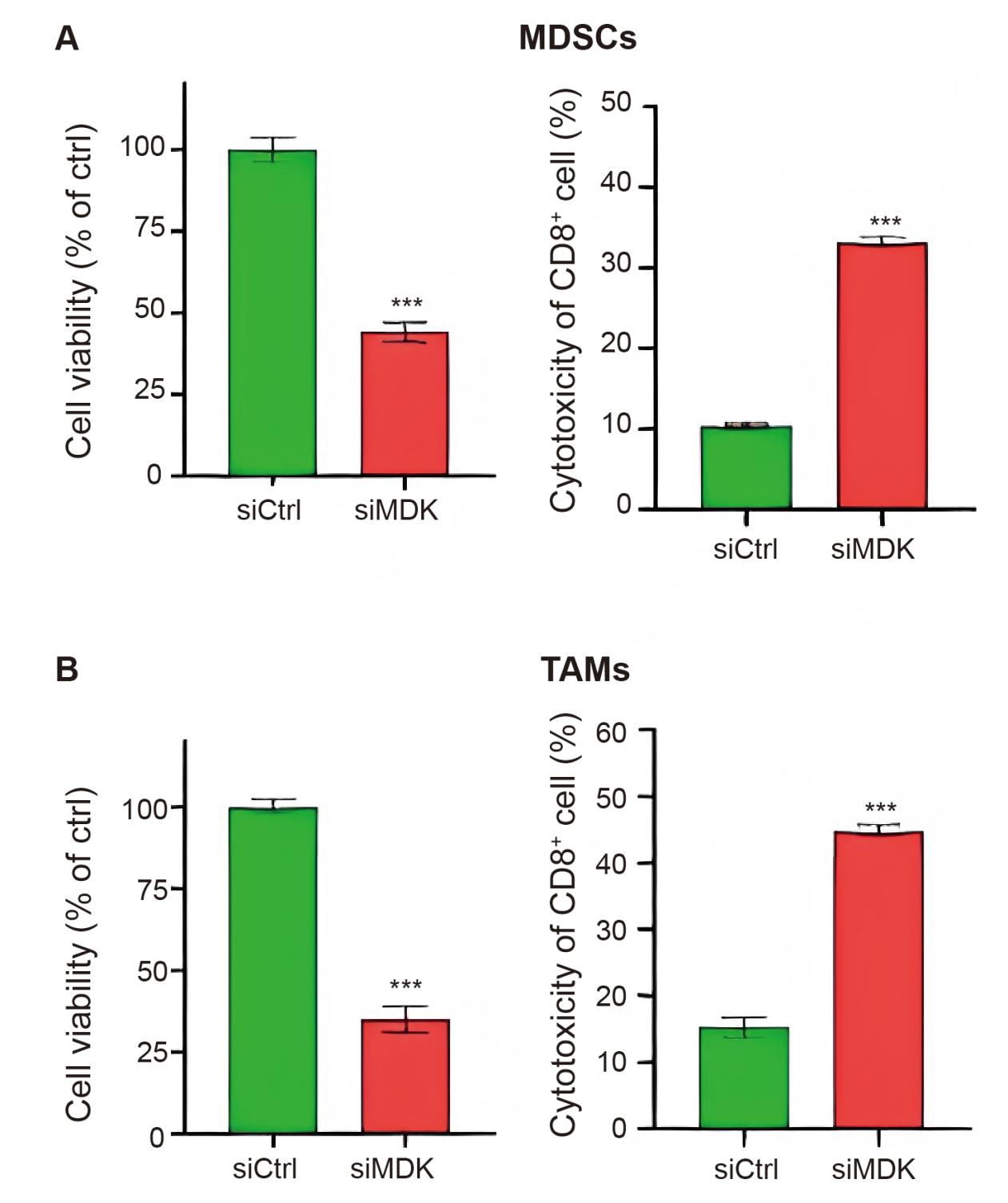
**

**Fig. S25. CD8^+^ T-cell cytotoxicity to tumor cells was evaluated by LDH assay**


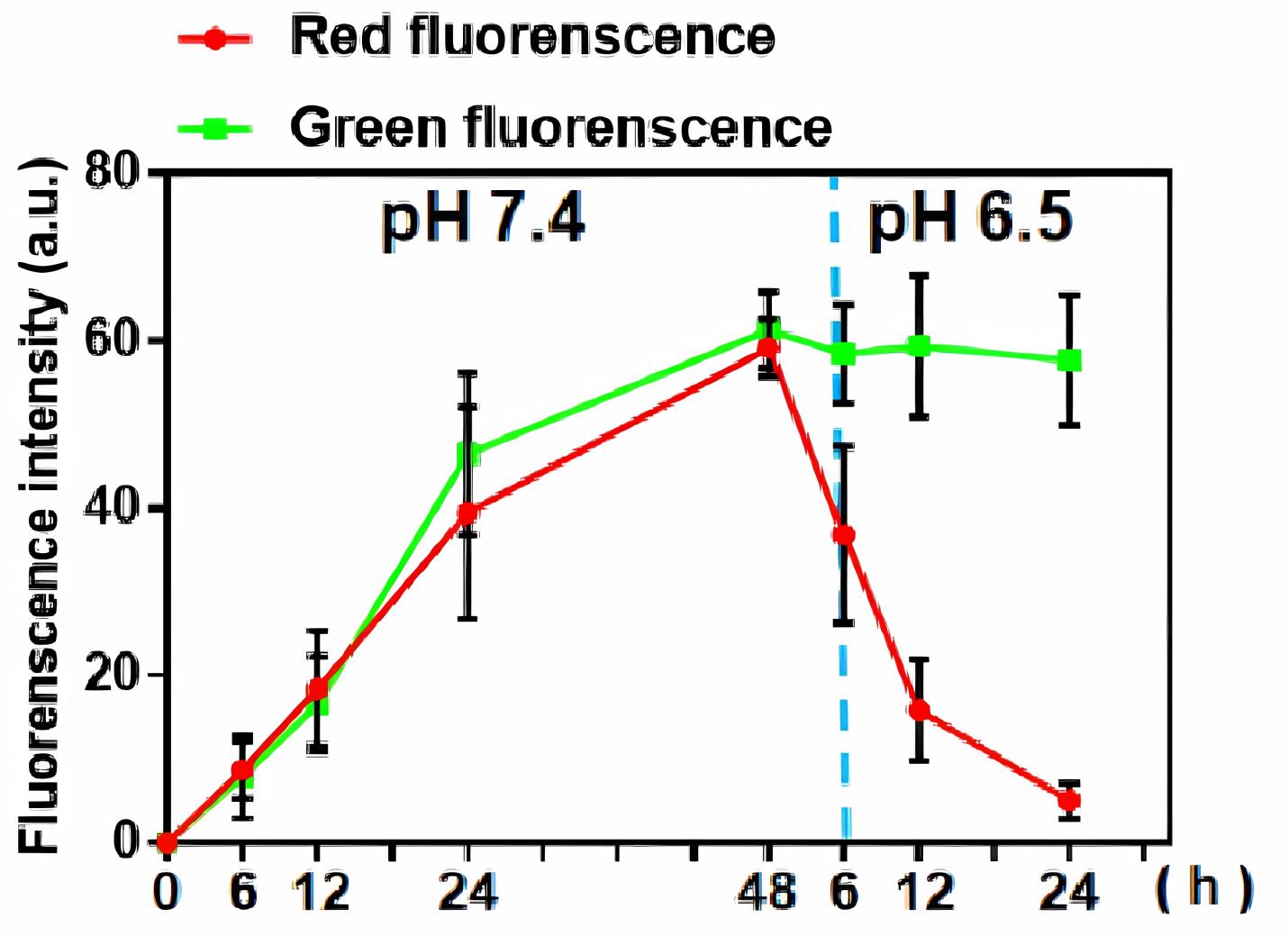


**Fig. S26. T cell binding of nanomedicine was observed in vitro by laser confocal microscopy (CLSM). aPD-1 (Green Alexa Fluor 488); MDK-siRNA (Nile Red hydrophobic red fluorescence).**


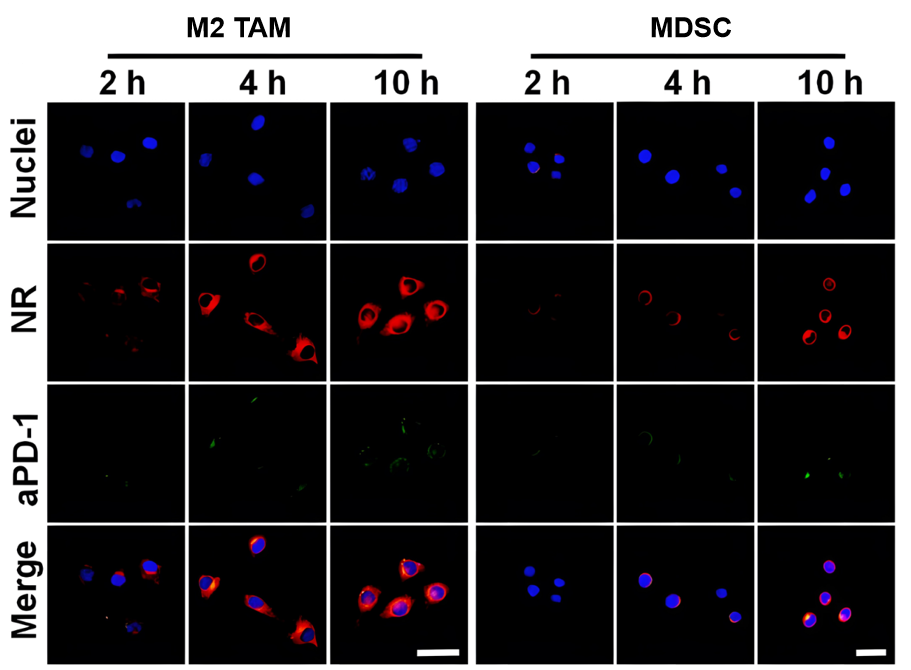


**Fig. S27. Nanodrug uptake by M2, TAM and MDSC observed by CLSM (pH 6.5)**


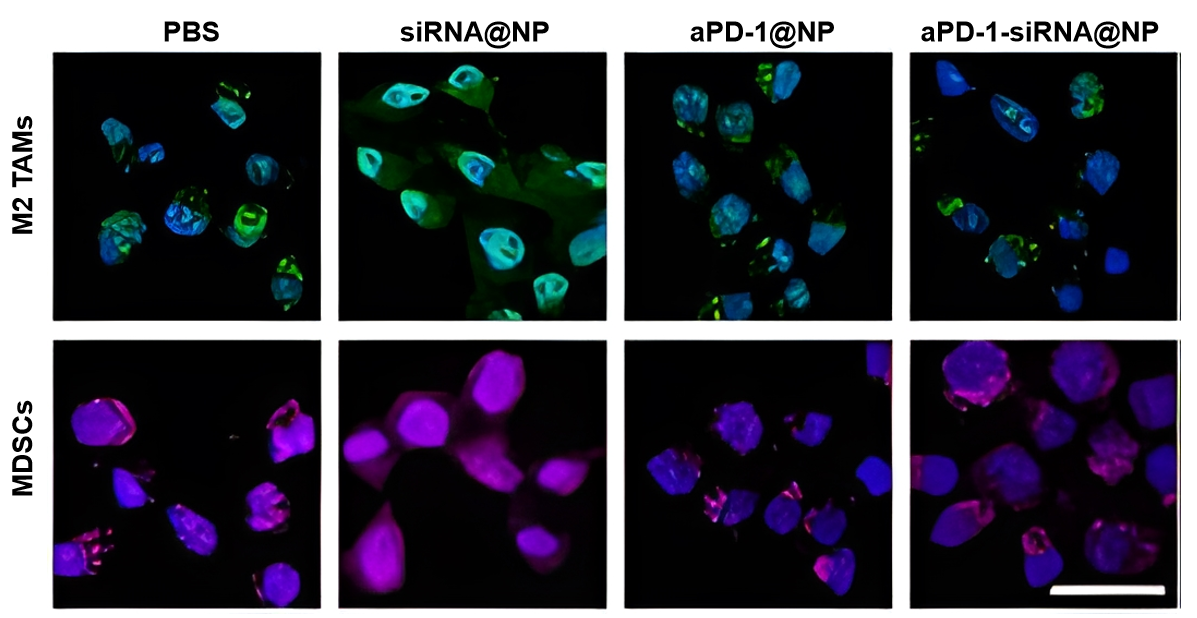


**Fig. S28. Nanomaterials inhibit NF-κB phosphorylation and PD-L1 expression**


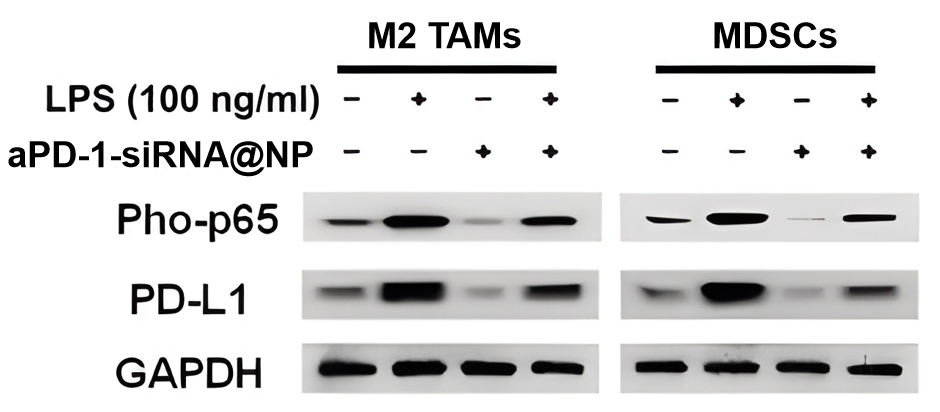
**Fig. S29. Nanomaterials inhibit NF-κB phosphorylation and PD-L1 expression**


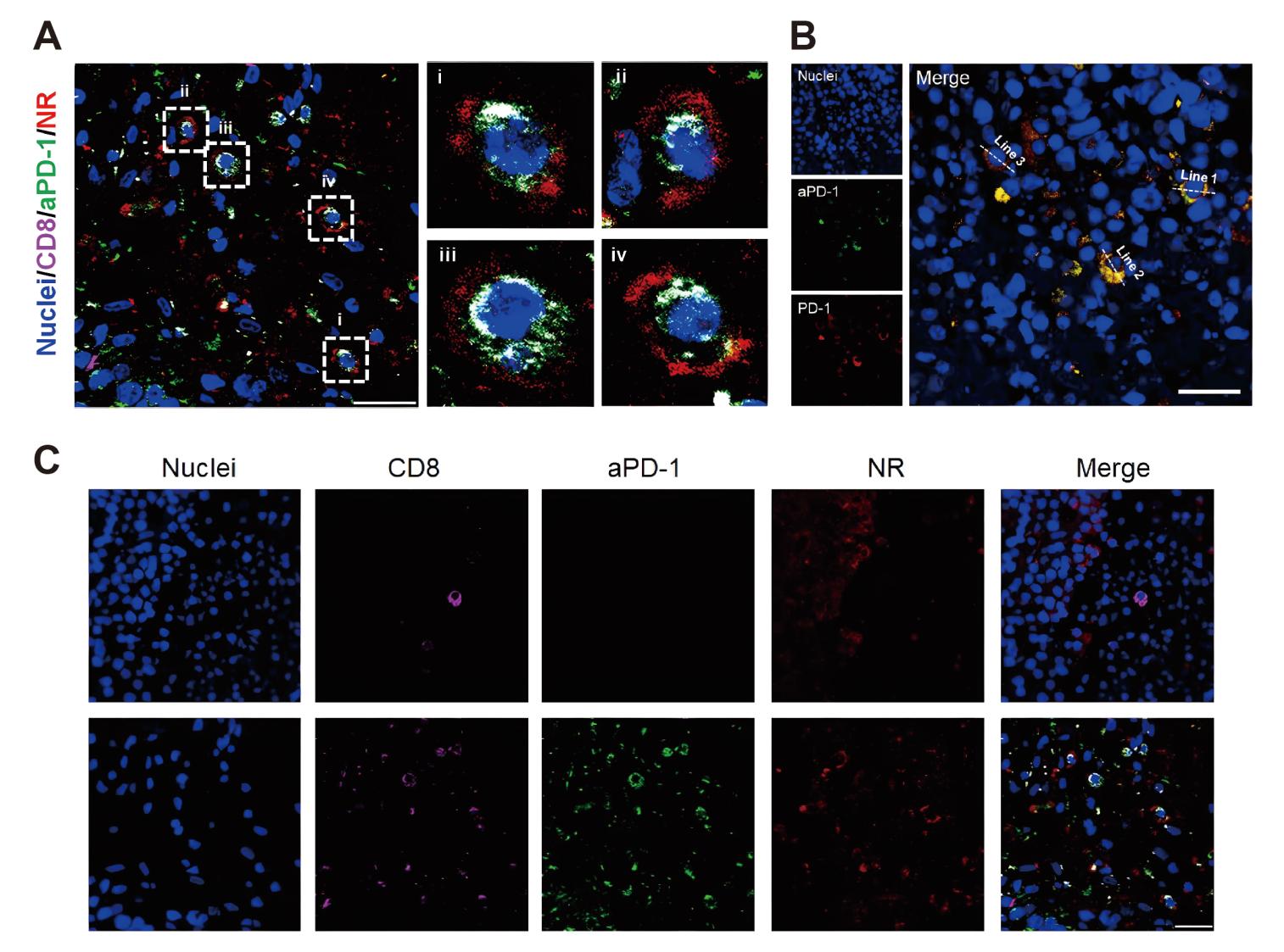


**Fig. S30. Nanomaterials inhibit NF-κB phosphorylation and PD-L1 expression**

**
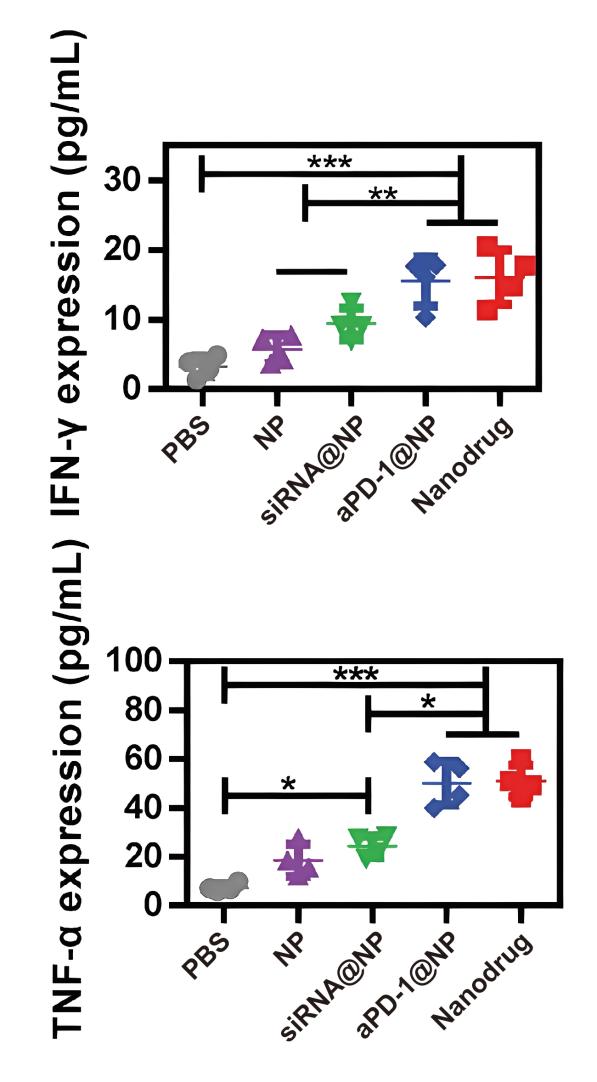
**

**Fig. S31. INF-γ and TNF-α by ELISA assay in tumor tissues of mice receiving treatments**


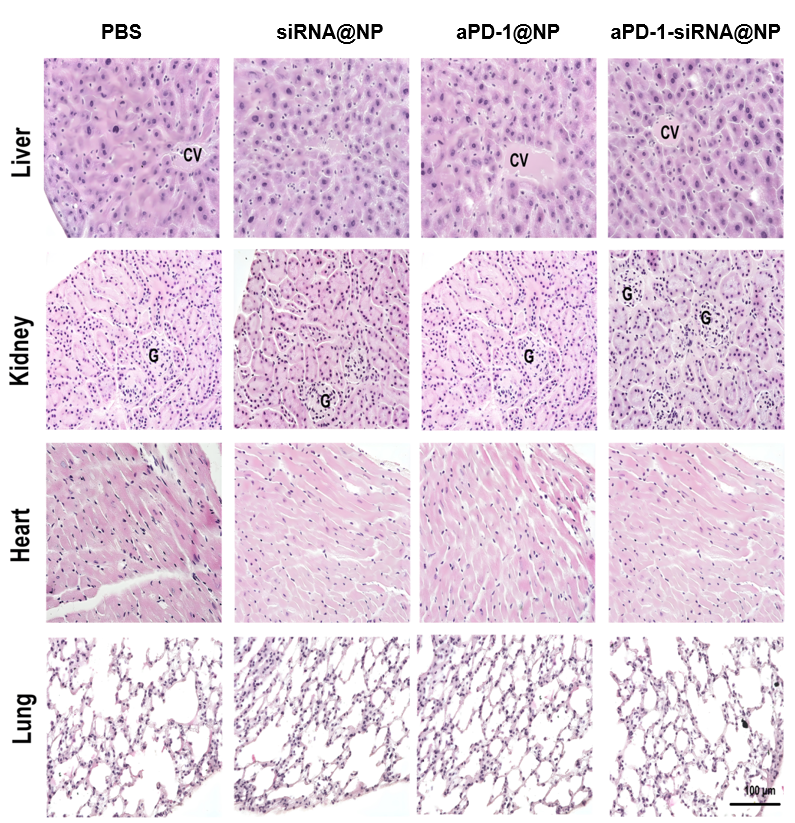


**Fig. S32. Biosafety of nanomedicine**

**Table S1. Pharmacokinetic parameters of the nanodrug following intravenous administration to mice (siMDK dose=0.5 mg/Kg, N=3, average ± standard deviation).**

| **Pharmacokinetic parameter** | **aPD-1-siRNA@NP** | **siRNA** |
| --- | --- | --- |
| t1/2 (h) | 9.73±0.53 | 8.08±0.31 |
| V (mL) | 1.00±0.11 | 1.08±0.24 |
| CL (mL/h) | 0.07±0.015 | 0.09±0.019 |
| AUC 0-t (%ID. h/mL) | 1147.21±298 | 944.68±175.11 |
| AUC 0-inf (%ID. h/mL) | 1400.45±331.5 | 1083.03±227.1 |
| AUMC (%ID. h2/mL) | 19653.03±981.9 | 12631.66±636.7 |
| MRT (h) | 14.03±2.8 | 11.66±1.95 |

t_1/2_, half-life time in the blood circulation; V, volume of distribution; CL, clearance; AUC 0-t, area under the circulation time curve from zero to end time points; AUC 0-inf, area under the

circulation time curve from zero to infinity time; AUMC, area under the moment curve; MRT, mean residence time.
